# Three-Dimensional Human iPSC-Derived Artificial Skeletal Muscles Model Muscular Dystrophies and Enable Multilineage Tissue Engineering

**DOI:** 10.1016/j.celrep.2018.03.091

**Published:** 2018-04-17

**Authors:** Sara Martina Maffioletti, Shilpita Sarcar, Alexander B.H. Henderson, Ingra Mannhardt, Luca Pinton, Louise Anne Moyle, Heather Steele-Stallard, Ornella Cappellari, Kim E. Wells, Giulia Ferrari, Jamie S. Mitchell, Giulia E. Tyzack, Vassilios N. Kotiadis, Moustafa Khedr, Martina Ragazzi, Weixin Wang, Michael R. Duchen, Rickie Patani, Peter S. Zammit, Dominic J. Wells, Thomas Eschenhagen, Francesco Saverio Tedesco

**Affiliations:** 1 Department of Cell and Developmental Biology, University College London, London WC1E 6DE, UK; 2 Department of Experimental Pharmacology and Toxicology, University Medical Center Hamburg Eppendorf (UKE), 20246 Hamburg, Germany; 3 DZHK (German Centre for Cardiovascular Research), partner site Hamburg/Kiel/Lübeck, Germany; 4 Randall Centre for Cell and Molecular Biophysics, King’s College London, London SE1 1UL, UK; 5 Department of Comparative Biomedical Sciences, Royal Veterinary College, London NW1 0TU, UK; 6 Institute of Neurology, University College London, London WC1N 3BG, UK; 7 The Francis Crick Institute, London NW1 1AT, UK

**Keywords:** skeletal muscle, pluripotent stem cells, iPS cells, myogenic differentiation, tissue engineering, disease modeling, muscular dystrophy, organoids

## Abstract

Generating human skeletal muscle models is instrumental for investigating muscle pathology and therapy. Here, we report the generation of three-dimensional (3D) artificial skeletal muscle tissue from human pluripotent stem cells, including induced pluripotent stem cells (iPSCs) from patients with Duchenne, limb-girdle, and congenital muscular dystrophies. 3D skeletal myogenic differentiation of pluripotent cells was induced within hydrogels under tension to provide myofiber alignment. Artificial muscles recapitulated characteristics of human skeletal muscle tissue and could be implanted into immunodeficient mice. Pathological cellular hallmarks of incurable forms of severe muscular dystrophy could be modeled with high fidelity using this 3D platform. Finally, we show generation of fully human iPSC-derived, complex, multilineage muscle models containing key isogenic cellular constituents of skeletal muscle, including vascular endothelial cells, pericytes, and motor neurons. These results lay the foundation for a human skeletal muscle organoid-like platform for disease modeling, regenerative medicine, and therapy development.

## Highlights

• Human iPSC-derived 3D artificial muscles show features of normal skeletal muscle
• Multiple muscular dystrophy iPSC lines can be differentiated in 3D artificial muscles
• Artificial muscle constructs model severe, incurable forms of muscular dystrophy
• Isogenic vascular-like networks and motor neurons develop within artificial muscles

## Graphical Abstract

**Figure:**
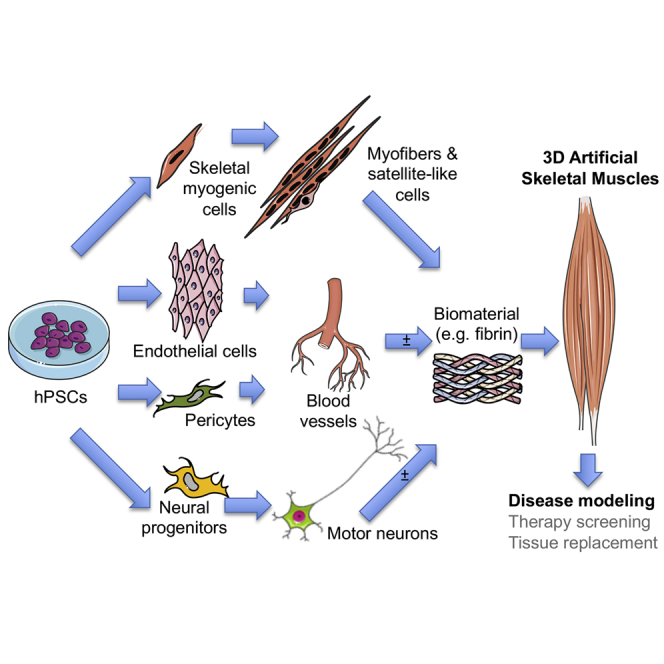

## Teaser

Maffioletti et al. generate human 3D artificial skeletal muscles from healthy donors and patient-specific pluripotent stem cells. These human artificial muscles accurately model severe genetic muscle diseases. They can be engineered to include other cell types present in skeletal muscle, such as vascular cells and motor neurons.

## Introduction

Skeletal muscle is the most abundant human tissue, and it is responsible for movement, posture, temperature control, and various metabolic functions. It is composed of aligned multinucleated myofibers, and its repair and regeneration rely on resident stem or progenitor cells, of which satellite cells are the best characterized (Tedesco et al., 2010). Nonetheless, impaired muscle regeneration occurs in acute or chronic conditions, such as significant trauma (Grogan et al., 2011), or incurable inherited disorders, such as muscular dystrophies (Mercuri and Muntoni, 2013). Artificial human skeletal muscles would provide an invaluable tool to study pathological mechanisms, test potential therapeutics, and develop tissue replacement protocols. Use of a similar approach in other tissues has proved transformational for drug development and regenerative medicine by means of organoid technology (Fatehullah et al., 2016, Lancaster and Knoblich, 2014).

Although several studies have reported methods to engineer rodent skeletal muscle tissue (Carosio et al., 2013, Corona et al., 2014, Huang et al., 2005, Juhas et al., 2014, Machingal et al., 2011, Shandalov et al., 2014, VanDusen et al., 2014), fewer groups have used human cells (Chiron et al., 2012, Fuoco et al., 2015, Madden et al., 2015, Powell et al., 1999, Quarta et al., 2017, Tchao et al., 2013). Apart from a recent study on healthy donor human pluripotent stem cells (hPSCs) for muscle tissue engineering (Rao et al., 2018), most studies used primary human cells from invasive muscle biopsies, facing hurdles such as poor cell availability, limited cell expansion potential, and exhaustion of differentiation ability. In addition, although there is an increasing need to develop clinically relevant multilineage patient-specific models (Giacomelli et al., 2017), no such isogenic human skeletal muscle model has been derived to date. These obstacles hinder the translational potential of these platforms for muscle diseases.

To overcome these limitations, here we have exploited the virtually unlimited proliferative capacity and controllable differentiation of human embryonic stem cells (hESCs) and human induced pluripotent stem cells (hiPSCs) (referred to collectively as hPSCs) (Inoue et al., 2014) to produce 3D artificial skeletal muscle constructs for complex muscle disease modeling. hPSCs were induced to skeletal myogenesis within a biocompatible hydrogel using established protocols (Caron et al., 2016, Maffioletti et al., 2015, Tedesco et al., 2012). Artificial muscles could be made using hiPSCs from Duchenne, limb-girdle type 2D, and LAMIN A/C (*LMNA*)-related muscular dystrophies. Our 3D platform modeled cellular hallmarks of *LMNA*-related muscular dystrophies with high fidelity. Finally, essential cell types present in muscle tissue, such as vascular cells and motor neurons, were derived from the same hiPSC source to generate isogenic multilineage muscle constructs.

## Results

### 3D Artificial Muscles Can Be Generated from Multiple Healthy and Dystrophic hPSC Lines

**Figure 1:**
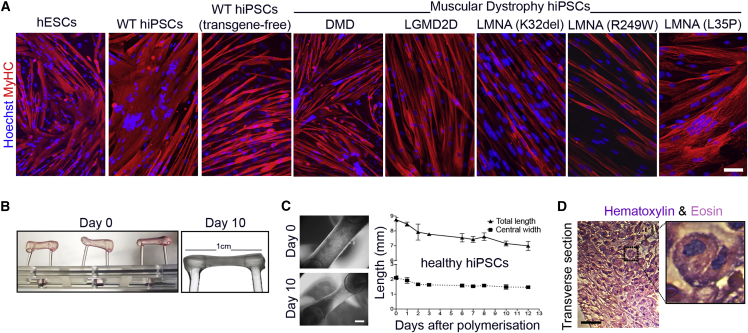
Differentiation of Multiple hPSC Lines into Skeletal Myotubes and Remodeling of Fibrin Hydrogels upon 3D Culture (A) Immunofluorescence analysis for myosin heavy chain (MyHC) in standard monolayer cultures of differentiated hESC- and hiPSC-derived cells (DMD, Duchenne muscular dystrophy; LGMD2D, limb-girdle muscular dystrophy type 2D; *LMNA*, skeletal muscle laminopathies, specific mutations listed). (B) Side-view of the 3D culture platform with freshly polymerized gels at day 0 (left) and day 10 (right) of culture containing hiPSC-derived myogenic cells. (C) Representative phase contrast images of cellularized hydrogels after polymerization (day 0) and after 10 days in culture. Gels undergo remodeling, shorten, and thin in culture (graph). Mean ± SD, N = 2–4 per time point. (D) H&E staining of a transverse hydrogel sections after 10 days of differentiation. Magnification: centronucleated myofibers. Scale bars: (A and D) 100 μm; (C) 1 mm.

**Figure 2:**
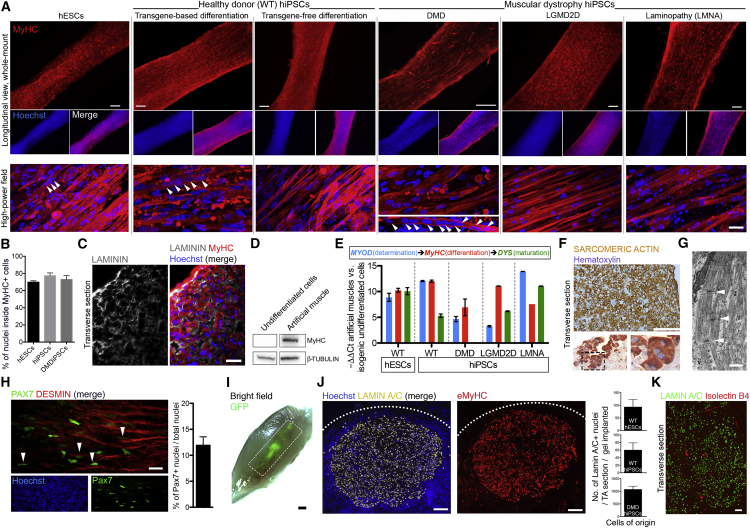
3D Artificial Skeletal Muscle Constructs Derived from Healthy and Dystrophic hPSCs (A) Whole-mount immunofluorescence for myosin heavy chain (MyHC) on muscle constructs derived from hESCs, WT hiPSCs (transgene-based and transgene-free differentiation protocols) and dystrophic hiPSCs (DMD, LGMD2D, and skeletal muscle LMNA) differentiated in 3D for 10 days. Nuclei are counterstained with Hoechst. Arrowheads: multinucleated myotubes. (B) Graph quantifying the proportion of MyHC^+^ cells using z stack confocal microscopy of three hPSC lines shown in (A). (C) Immunolabeling for LAMININ (extracellular matrix), MyHC, and nuclei (Hoechst) on DMD artificial muscles. (D) Western blot for MyHC (250 kDa) in undifferentiated and 3D differentiated iPSC-derived, inducible myogenic cells. β-tubulin: loading control (50 kDa). (E) qRT-PCR analysis of artificial muscles for myogenic markers. *DYSTROPHIN* (DYS) is absent from DMD-derived artificial muscles. N = 3 for all lines apart from *LMNA* mutant and LGMD2D hiPSCs, whose error bars represent intra-experimental replicates (n = 3). Values are normalized on *GAPDH* expression; ΔΔCt is calculated on the corresponding expression values of undifferentiated cells. (F) Immunohistochemistry for sarcomeric actin in DMD artificial muscles after 10 days of differentiation. (G) Transmitted electron microscopy images of DMD iPSC-derived artificial muscle showing sarcomeres (white arrowheads: z lines). (H) Immunofluorescence showing PAX7^+^ nuclei adjacent to DESMIN^+^ myofibers following transgene-free commitment and differentiation of hiPSCs in 3D for 14 days. The graph quantifies the percentage of PAX7^+^ nuclei within the hydrogels (a total of 5,341 nuclei across 10 random fields). (I) Bright-field image of a tibialis anterior (TA) muscle 1 week after implantation of artificial muscles generated using GFP^+^ myogenic cells. Dashed rectangle: grafted area. (J) Immunofluorescence showing engrafted human nuclei (LAMIN A/C^+^, left) corresponding to an area in a serial section with embryonic MyHC^+^ (eMyHC) fibers in transverse sections of a TA muscle 1 week after implantation. Right graphs show quantification of human nuclei from three healthy or dystrophic cell lines; N = 6, 2 mice/cell type; mean ± SD: hESCs 92 ± 30, hiPSCs 59 ± 19, DMD hiPSCs 1,068 ± 132. (K) Immunofluorescence of systemically delivered 594-conjugated IB4 isolectin (red) labeling endothelial cells within the implanted human artificial muscle (LAMIN A/C: human nuclei). Error bars: mean ± SD. Scale bars: (A) top 250 μm, bottom 25 μm; (C, F, and K) 100 μm; (G) 1 μm; (H) 20 μm; (I) 1 mm; (J) 200 μm. For additional information, see Figures S1 and S2.

To generate 3D human artificial skeletal muscles, hPSC-derived myogenic cells (healthy and dystrophic) (Figure 1A) were embedded in fibrin hydrogels and differentiated, adapting a cardiac tissue engineering platform (Hansen et al., 2010). Fibrin was polymerized from fibrinogen in molds between two flexible silicone posts providing continuous tension to the gel, sufficient to direct orientation of cells along the force axis (Figure 1B). Over 10 days, cells remodeled the matrix, generating a 7–8 mm long strip of tissue containing structures resembling skeletal myofibers (Figures 1C, 1D, and S1A). Both transgene-based (Maffioletti et al., 2015) and transgene-free (Caron et al., 2016) differentiation protocols produced muscle constructs (Figures 2A and S1B). Transgene-based muscle constructs were generally used due to the better alignment of myofibers, easier scalability of cultures, and experimental cost-effectiveness.

Immunolabeling of artificial muscles from hESCs and hiPSCs—healthy donor, Duchenne muscular dystrophy (DMD), limb-girdle muscular dystrophy type 2D (LGMD2D) (Figure S1C), and *LMNA*-related muscular dystrophies—showed homogeneous presence of myosin heavy chain (MyHC)^+^ multinucleated myotubes oriented along the force axis of the hydrogels (Figures 2A and 2B), as observed with primary human myoblasts within the same platform (Figure S1D). Transverse sections of artificial muscles revealed abundant MyHC^+^ muscle fibers surrounded by LAMININ^+^ extracellular matrix (Figure 2C). Western blot analysis confirmed production of MyHC protein (Figure 2D). Healthy and dystrophic muscle constructs also expressed markers of skeletal muscle determination and maturation (e.g., *MYOD* and *DYSTROPHIN*) (Figure 2E). Immunohistochemical staining highlighted proteins associated with skeletal muscle maturation, such as sarcomeric actin (Figure 2F). Electron microscopy revealed that muscle constructs displayed some degree of cytoskeletal organization into sarcomeres, the basic functional unit of striated myofibers (Figure 2G). Functional myotubes within the artificial muscles were detected by caffeine-induced calcium transients (Figure S1E).

Pax7 marks a population of self-renewing myogenic stem cells known as satellite cells (Tedesco et al., 2010). To assess whether our 3D platform generated and provided a niche for Pax7^+^ cells, we induced myogenic commitment and differentiation of transgene-free hiPSC-derived myogenic progenitors directly in hydrogels. This resulted in PAX7^+^ cells juxtaposed to myofibers, albeit with variability among the tested hiPSC lines (Figure 2H).

To further characterize hPSC-derived artificial muscles, they were implanted into immunodeficient mice. Tibialis anterior (TA) muscles of non-obese diabetic (NOD)-severe combined immunodeficiency (SCID)-gamma mice (NSG) (N = 24) were injured, and a strip of host tissue was replaced with GFP-expressing hPSC-derived artificial muscles. GFP^+^ implants were identifiable in explanted TA muscles at various time points (Figures 2I and S2A), suggesting successful engraftment. Transverse sections of implanted muscles showed engraftment of human cells, highlighted by the widespread presence of human LAMIN A/C^+^ nuclei (Figures 2J and S2B). LAMIN A/C^+^ areas colocalized with embryonic MyHC^+^ (eMyHC) fibers, demonstrating skeletal muscle generation *in vivo* (Figures 2J, S2C, and S2D). Engraftment was confirmed by expression of human muscle-specific transcripts in implanted muscles, and blood vessels within the implants were detected by immunolabeling for CD31 (Figures S2E–S2G). We further investigated vascularization of the artificial muscle by systemically injecting fluorescent isolectin into the mouse circulation before harvesting implanted muscles. Isolectin^+^ vessels were evident within the implant, confirming functional vascularization (Figure 2K).

Therefore, fibrin hydrogels under uniaxial tension stimulate efficient and aligned 3D skeletal myogenic differentiation of healthy and dystrophic hPSCs. Muscle constructs recapitulate distinctive molecular, structural, and functional features of skeletal muscle and engraft in immunodeficient mice.

### hiPSC-Derived Artificial Skeletal Muscles Enable Disease Modeling of Skeletal Muscle Laminopathies

Organoids have great potential for disease modeling and drug development, so we examined whether our organoid-like, artificial skeletal muscle could model severe and incurable forms of muscular dystrophy. We also hypothesized that the 3D nature of our hydrogels would facilitate detection of pathological hallmarks less evident in standard bi-dimensional cultures. To investigate this, we examined artificial muscles generated from hiPSC derived from patients with muscular dystrophies caused by mutations in the *LMNA* gene. *LMNA* mainly encodes the A-type lamins, lamin A and lamin C (LAMIN A/C), nuclear envelope proteins that assemble with B-type lamins into the nuclear lamina, providing structural support and regulating gene expression (Worman, 2012). *LMNA* mutations cause a plethora of diseases called laminopathies, of which three forms affect skeletal muscle (Maggi et al., 2016): limb-girdle muscular dystrophy type 1B (LGMD1B), autosomal dominant Emery-Dreifuss muscular dystrophy 2 (EDMD2), and *LMNA*-related congenital muscular dystrophy (L-CMD). Abnormalities in nuclear morphology are a key histological feature of skeletal muscle laminopathies (Park et al., 2009), and using hiPSC-based modeling could provide a unique, non-invasive tool to address open questions, such as challenging genotype-phenotype correlations, and develop new therapeutics (Scharner et al., 2015).

**Figure 3:**
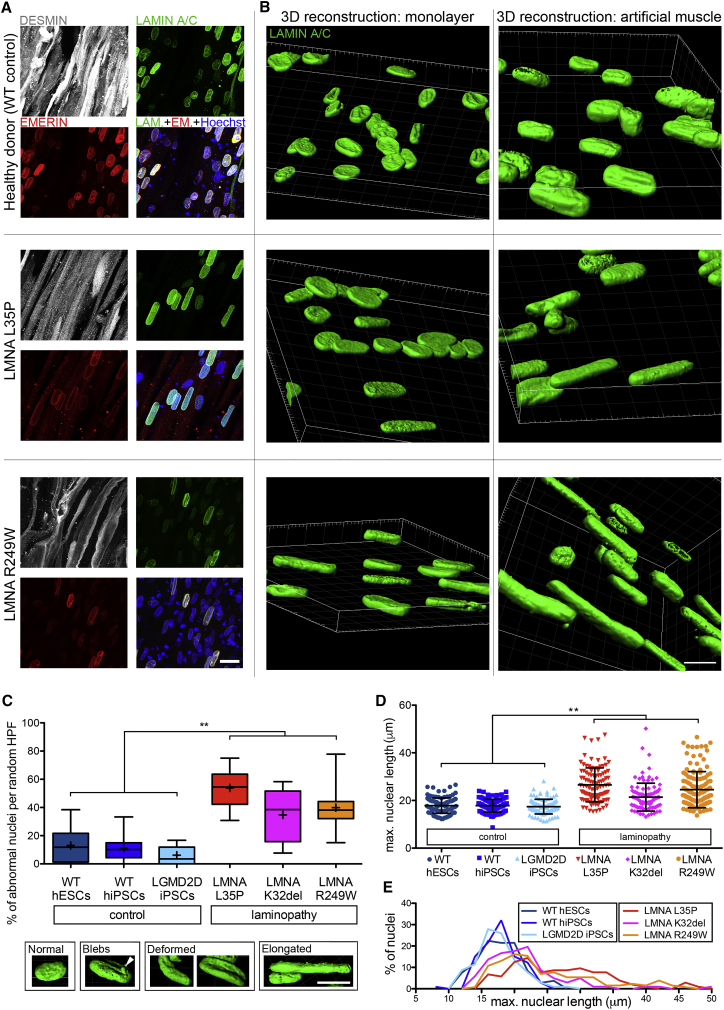
hiPSC-Derived Artificial Skeletal Muscles Model Skeletal Muscle Laminopathies (A) Confocal (z stacks merge) whole-mount immunofluorescence for DESMIN (myotubes), LAMIN A/C, and EMERIN (nuclear lamina) on hiPSC-derived (healthy and *LMN*A mutant) artificial muscles. Hoechst: nuclei. (B) Comparison of confocal 3D nuclear reconstructions of the same iPSC lines shown in (A) differentiated as monolayer cultures (left) versus 3D artificial muscle constructs (right). Nuclei are immunolabeled for LAMIN A/C. (C) Box and whiskers graph quantifying nuclear abnormalities in hiPSC-derived artificial muscle generated from 3 patients affected by *LMNA*-related muscular dystrophies (red color shades) versus 3 control donors (blue color shades). LGMD2D artificial muscles are included as negative control, because they do not have nuclear abnormalities. Lower panel: representative images of 3D-reconstructed nuclei used to score laminopathy versus control muscles in the graph. ^∗∗^p = 0.0022, Mann-Whitney U test. n = 6 in CTRL group and 6 in *LMNA* mutant group (3 cell populations per group in 2 independent experiments). A minimum of 45 nuclei/hydrogel/experiment across 8 random high-power fields were scored. Boxes, 25^th^ to 75^th^ percentiles; horizontal line inside, median; +, mean; whiskers, min to max values. (D) Scatter dot plot of the specific length of the nuclei scored in (C). ^∗∗^p = 0.0022, Mann-Whitney U test. n = 6 in CTRL group and 6 in *LMNA* mutant group (3 cell populations per group in 2 independent experiments). Each symbol is one nucleus. Error bars: mean ± SD. (E) Distribution plot of the graph in (D). Scale bars: (A and B) 15 μm; (C) 10 μm. For additional information, see Table S1 and Videos S1 and S2.

3D artificial muscles were made by differentiating three *LMNA* mutant hiPSCs from patients with skeletal muscle laminopathies, referred to by their mutation as *LMNA* L35P, R249W, and K32del (Figures 1A, 2A, and 3A). 3D nuclear reconstruction of mutant *LMNA* cells differentiated in artificial muscles highlighted features less prominent in standard monolayer cultures (Figure 3B). This prompted us to quantify nuclear abnormalities, including elongation, deformities, and presence of blebs (Figure 3C), in *LMNA* mutant hiPSC-derived artificial muscles. For controls, we used wild-type (WT) hPSCs and LGMD2D hiPSCs (Figures 1A and 2A), which have not been reported to have dysmorphic nuclei (Kirschner and Lochmüller, 2011). As expected, nuclei in artificial muscle derived from control cells did not display significant nuclear abnormalities. In contrast, all mutant *LMNA* artificial muscles showed a significant proportion of cells with nuclear aberrations (p = 0.0022, N = 6) (Figure 3C; Table S1; Videos S1 and S2).

Nuclear elongation was a predominant abnormality, in line with biopsy-derived primary myoblast 3D cultures (Bertrand et al., 2014). Measurements confirmed that laminopathic muscle constructs contained significantly elongated nuclei compared to control artificial muscles (p = 0.0022) (Figures 3D and 3E), supporting using this outcome measure in future therapy screening platforms. Thus, hiPSC-derived artificial muscles recapitulate cellular hallmarks of skeletal muscle laminopathies with high fidelity and are amenable to model severe muscle disorders.

### Increasing Histological Complexity: Multilineage hiPSC-Derived 3D Artificial Skeletal Muscles

**Figure 4:**
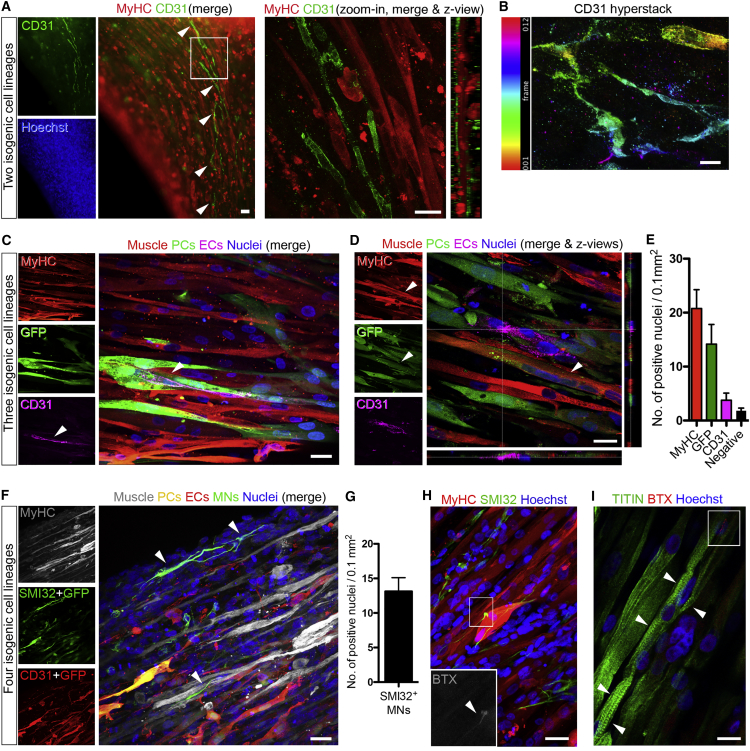
Multilineage Artificial Muscles Containing Isogenic hiPSC-Derived Vascular Cells and Motor Neurons (A) Left panel: whole-mount immunofluorescence of an artificial muscle containing a self-organized isogenic network of hiPSC-derived CD31^+^ endothelial cells (arrowheads). Right panel: higher-magnification confocal image of the boxed area showing lateral z views. (B) Hyper-stack image (12 frames) processed with color-coding on CD31 staining (ImageJ) highlighting the 3D structure of the endothelial network. Frame thickness: 2 μm. (C) Confocal images of whole-mount immunolabeling of a multilineage construct containing GFP^+^ pericytes (PCs), displaying coexistence of myofibers (MyHC), ECs (CD31), and PCs. Arrowheads: CD31^+^ ECs juxtaposed to PCs. (D) Confocal image showing an additional example of a multilineage construct as in (C) with lateral z views. Arrowhead indicates a MyHC^+^ and GFP^+^ multinucleated myotube (see Discussion). (E) Quantification of confocal images of tri-lineage artificial muscles showing the average number of MyHC^+^ (muscle), GFP^+^ (PCs), and CD31^+^ (ECs) nuclei per 0.1 mm^2^ field. Error bars: SEM. n = 10 images. (F) Confocal immunofluorescence panel of multilineage 3D artificial muscle derived from WT hiPSCs containing isogenic myofibers, vascular cells (ECs and GFP^+^ PCs), and motor neurons (SMI32). Color-coded combination enabled discrimination of the four cell types based upon the color of the merge. Arrowheads highlight two motor neurons showing multiple axon-like processes. (G) Quantification of the motor neurons (MNs) in quadruple lineage cultures shown in (F). Error bars: SEM. n = 7 images. (H) Confocal immunofluorescence of a DMD artificial muscle construct containing non-isogenic WT SMI32+ motor neurons and showing alpha-bungarotoxin (BTX)^+^ acetylcholine receptors (zoom in). (I) Confocal immunofluorescence of a sister construct of (H) with aligned, multinucleated myofibers with sarcomeric TITIN+ striations (arrowheads) and BTX^+^ acetylcholine receptors (red signal in white box). Scale bars: (A and B) 25 μm; (C–F, H, and I) 10 μm. For additional information, see Figure S3.

Skeletal muscle tissue also contains non-muscle support cell types, such as vascular endothelial cells (ECs) and pericytes (PCs), which permit blood perfusion while controlling homeostasis of the muscle stem cell compartment (Christov et al., 2007). Therefore, adding ECs and PCs to artificial muscles could generate a more physiologically relevant model *in vitro* and improve survival of larger constructs *in vivo*, which require prompt vascularization to prevent hypoxia-induced cell death (Criswell et al., 2013, Gholobova et al., 2015, Koffler et al., 2011, Levenberg et al., 2005). To achieve these aims, we first derived isogenic ECs and PCs from the same hiPSCs used for myogenic differentiation (Figure S3A) (Orlova et al., 2014). We then combined isogenic hiPSC-derived ECs, PCs, and myogenic cells within hydrogels under tension and tested media that supported growth and differentiation of those lineages. After 10 days in culture, long (up to 0.9 mm) CD31^+^ vessel-like formations coexisted in the same 3D environment close to isogenic myofibers (Figure 4A). The 3D nature of EC networks was visualized through hyper-stack images processed with depth color-coding for CD31 immunolabeling (Figure 4B). Due to the absence of unequivocal PC-specific markers (Armulik et al., 2011), hiPSC-derived PCs were transduced with a lentivirus encoding for GFP to allow their detection within artificial muscles. Immunofluorescence demonstrated alignment and coexistence of myofibers, ECs, and PCs within the same 3D environment (Figures 4C–4E). *In vivo* studies of muscle function in immunodeficient mice suggested enhanced force recovery only in muscles receiving multicellular versus single-lineage (i.e., only myofibers) artificial muscle implants (Figure S3B).

Another key cell type for skeletal muscle is the spinal motor neuron. Deriving primary human motor neurons is challenging; however, several protocols are available to differentiate them from hPSCs (Patani, 2016), including their coculture with primary myotubes (Steinbeck et al., 2016). Therefore, developing an isogenic human muscle-motor neurons platform would model neuromuscular disorders in a personalized fashion. To this aim, we differentiated hiPSCs into neural progenitors (Stacpoole et al., 2011) and further adapted our tri-lineage culture system to enable differentiation of neural precursors into motor neurons. We obtained stable 3D artificial muscle constructs containing four distinct isogenic cell types, i.e., myofibers, ECs, PCs, and SMI32+ cells with long axon-like processes resembling motor neurons spreading from hiPSC-derived neurospheres placed above the hydrogels (Figures 4F and 4G).

We next investigated generation of neuromuscular junctions through muscle-motor neuron bi-lineage models. Interactions between the two cell types were optimized by employing a differentiation paradigm that generates a highly enriched motor neuron population (Figure S3C) (Hall et al., 2017) and allows seeding of single-cell neural precursors within the hydrogel. DMD muscle constructs were made containing WT motor neurons, which showed alpha-bungarotoxin^+^ acetylcholine receptors and striated myofibers with the sarcomeric protein Titin (Figures 4H and 4I), indicating a positive effect on myofibers’ maturation exerted by motor neurons. Thus, we generated complex, 3D, multilineage, artificial skeletal muscle models from hiPSCs.

## Discussion

Use of hPSCs for tissue engineering and complex disease modeling is expanding, and exciting results have been obtained with hiPSC-derived organoids (Passier et al., 2016). Here we show that 3D constructs resembling skeletal muscle tissue can be generated by differentiating healthy donor and disease-specific hPSCs within fibrin hydrogels under unidirectional tension. We have generated artificial muscles from patients affected by severe forms of muscle diseases with different genetic inheritance, namely, Duchenne (X-linked), LGMD2D (autosomal recessive), and *LMNA*-related (autosomal dominant) muscular dystrophies. Our hiPSC-derived 3D platform recapitulated nuclear abnormalities characteristic of *LMNA*-related muscular dystrophies. Nuclear elongation was the most prominent abnormality, consistent with reports using *LMNA* mutant mice and primary human myoblasts (Bertrand et al., 2014, Nikolova et al., 2004), supporting the high fidelity of our 3D platform for modeling skeletal muscle laminopathies. We also provide proof of principle of hPSC-derived artificial muscle engraftment, laying the foundation for *in vivo* modeling and drug testing in humanized dystrophic muscles.

A major advantage of using hPSCs for muscle bioengineering is the ability to derive different cell types from the same cellular source, and here we provide evidence of generation of isogenic, multilineage, hPSC-derived artificial skeletal muscles. Although our data and published work (Rao et al., 2018) indicate that skeletal muscle properties can be observed in bundles of hPSC-derived myofibers, our results further indicate that maturation of fully functional artificial muscles might require contribution from other cellular lineages, such as vascular cells and motor neurons (Christov et al., 2007, Ecob-Prince et al., 1986, Kostallari et al., 2015, Martin et al., 2015). This could be particularly relevant for *in vitro* studies of non-muscle-specific defects in muscle disorders (Dabiré et al., 2012, Palladino et al., 2013) or to predict off-target effects of skeletal muscle-directed therapeutics. Artificial muscles containing vascular cells will likely improve engraftment upon implantation *in vivo*, via rapid anastomosis with the host circulation, in line with improved engraftment of vascularized muscle constructs derived from primary myoblasts (Perry et al., 2017, Quarta et al., 2017). Moreover, multilineage artificial muscles could also provide insights into human muscle regeneration dynamics, because we observed generation of GFP^+^ myofibers in constructs in which only PCs had been transduced with a GFP-encoding lentiviral vector (Figure 4D). This suggests myogenic potential of PCs or their recruitment from differentiating muscle, similar to that described in mice with lineage tracing experiments (Dellavalle et al., 2011).

This platform can be further engineered to include other pluripotent derivatives, such as different muscle interstitial cells (Tedesco et al., 2017). Nonetheless, bioengineering an all-hPSC-derived muscle will require highly specialized culture conditions, potentially with a combination of transgene-based (e.g., Darabi et al., 2012, Tedesco et al., 2012) and transgene-free (e.g., Caron et al., 2016, Chal et al., 2015) differentiation methods, because culture conditions to maintain non-myogenic cells might interfere with transgene-free, small molecule-based myogenic differentiation of hPSCs. Further optimization of artificial muscles includes improvement of maturation via chemical, electrical or optical stimulation and scaling down for high-throughput screening. Moreover, culture in autologous fibrinogen (de la Puente and Ludeña, 2014) would enable a highly personalized platform. Finally, human muscle models would reduce laboratory animal use for toxicity testing. In conclusion, this hPSC-derived artificial skeletal muscle platform could bring together regenerative medicine and drug development under the same translational technology, advancing knowledge on the pathogenesis and development of therapies for muscle diseases.

## Experimental Procedures

### Generation of 3D Skeletal Muscle Constructs

15 hPSC lines (1 hESC line + 14 hiPSC lines) and 1 primary human myoblast line were used. Hydrogels were produced as published (Hansen et al., 2010) and according to the manufacturer’s instructions (EHT Technologies, Hamburg). 10^6^ myogenic cells pre-treated with ROCK inhibitor (10 μM; 1–2 hr) were used per construct (total volume: 120 μL). Artificial muscles were cultured at 37°C with 5% CO_2_ supplementing the medium with 33 μg/μL aprotinin (Sigma, A3428) to prevent fibrinogen degradation. To induce myogenic differentiation, 1 μM 4-OH tamoxifen was added 48 hr after polymerization in proliferation medium and then 24 hr later in differentiation medium. For transgene-free myogenic differentiation (Genea Biocells), progenitors were cultured for 7 days in commitment medium before cells were combined with fibrin, switching to differentiation medium 2 days later. To generate PAX7^+^ cells, myogenic commitment was induced for 2 days in standard monolayer culture conditions and then continued in 3D for 5 days before switching to differentiation medium; hydrogels were cultured for a total of 14 days. Pax7^+^ cells were observed in 1 of 3 lines (NCRM1 iPSCs).

Triple-lineage constructs were made with the same method using a mix of 70% myogenic cells (7 × 10^5^) and 30% vascular cells (6 × 10^4^ ECs and 2.4 × 10^5^ PCs). Muscles were cultured in a 1:1 mix of human iPSC-derived mesoangioblast-like inducible myogenic cells (HIDEM) proliferation medium (Maffioletti et al., 2015) and endothelial medium A (EC-SFM [endothelial cell-serum-free basal medium]; 1% platelet-poor, plasma-derived serum; 30 ng/mL vascular endothelial growth factor [VEGF]; 20 ng/mL basic fibroblast growth factor [bFGF]) for 48 hr and then in a 1:1 mix of HIDEM differentiation medium (Maffioletti et al., 2015) and endothelial medium B (Lonza, CC-3162) after the second tamoxifen administration. Muscles were cultured for 10 days at 37°C with 5% CO_2_, changing the medium every other day. Human fibrinogen was kindly provided by Prof. H. Redl (LBG, Vienna).

Constructs containing four lineages (muscle + ECs + PCs + motor neurons) were made as described earlier; after 1 hr of polymerization, 6 neurospheres (neural progenitor cells [NPCs]) were decanted on top of the hydrogels using 10 μL of fibrin. After the second hour of polymerization, hydrogels were placed in media containing an equal ratio of Iscove's Modified Dulbecco's Medium (IMDM)-based HIDEM proliferation medium, EC-SFM (Orlova et al., 2014), and a chemically defined medium (Stacpoole et al., 2011), supplemented with heparin (5 μg/mL) and retinoic acid (0.1 μM). NPC differentiation to motor neurons was achieved through supplementation of the media with retinoic acid for 7 days and then with retinoic acid and purmorphamine (1 μM) until fixation at day 14.

To produce hydrogels containing myofibers (70%) and single-cell motor neurons (30%) (derived as per Hall et al., 2017), constructs were cultured for 48 hr using a 1:1 mix of HIDEM proliferation medium with 1% fetal bovine serum (FBS) and neural precursor medium (Shi et al., 2012). The same mix was supplemented with 4-OH tamoxifen and 0.1 μM γ-secretase inhibitor (Sigma, L1790) to induce myogenic and neural differentiation. After 24 hr, medium was changed to administer the second tamoxifen pulse. Hydrogels were kept in culture for 15 days at 37°C with 5% CO_2_, changing the medium mix with 0.1 μM γ-secretase inhibitor every day. At day 5, the medium was supplemented with agrin (R&D Systems, 550-AG/CF) to promote neuromuscular junction formation. Agrin concentrations of 0.1, 0.5, and 1 nM were used over the 3 initial days, and the final concentration was kept until fixation of constructs. Additional information can be found in Supplemental Experimental Procedures.

### Analysis of Nuclear Abnormalities in *LMNA* Mutant Artificial Muscles

*LMNA* mutant iPSCs derived from three patients affected by skeletal muscle laminopathies (LGMD1B and L-CMD) were provided by Cellular Dynamics International (CDI; http://www.cellulardynamics.com) and Cure Congenital Muscular Dystrophy (CureCMD; http://www.curecmd.org). iPSCs were derived by CDI using episomal vectors from samples provided by CureCMD, which holds patients’ clinical information. Pluripotency was tested by CDI with a proprietary set of genes. *LMNA* mutant iPSCs had three heterozygous dominant mutations: p.K32del and p.L35P located in *LMNA* exon 1 and p.R249W in *LMNA* exon 4.

Hydrogels were fixed with 4% paraformaldehyde (PFA) for 3 hr at 4°C followed by 6 hr of blocking at 4°C (10% FBS, 1% BSA, and 0.5% Triton X-100 in 0.05 M Tris-buffered saline [TBS]) before immunolabeling with rabbit anti-Desmin (1:150) (Sigma, D8281), mouse anti-LAMIN A/C (1:100) (Novocastra NCL-LAM), and goat anti-Emerin (1:50) (Santa Cruz, sc8086) antibodies overnight at 4°C in TBS, 1% BSA, and 0.5% Triton X-100. The next day, hydrogels were washed with TBS 6 times hourly and incubated overnight with Hoechst 33342 (Sigma, B2261) plus species-specific secondary antibodies (Alexa Fluor 488, 546, and 647) (Thermo Fisher Scientific). The following day, hydrogels were washed 6 times with TBS and embedded in mounting medium (Dako, S3023A) on glass slides. A confocal microscope (Leica, SPE2) was used for imaging, using 95× magnification to take 5 to 9 z stacks (step size: 0.5 μm) of randomly selected regions (final thickness: 12–82 μm). The z stacks were 3D reconstructed and analyzed based on LAMIN A/C immunolabeling using Imaris 8.4.1 software (Bitplane). Nuclei were analyzed by scoring the number of abnormalities per field and by measuring major axis length. Oval or slightly elongated nuclei were scored as normal. Three *LMNA* mutant patients and three controls were quantified. A minimum of 45 nuclei per hydrogel per experiment across 8 random fields was scored. Reproducibility was validated by 3 independent operators (one researcher was blinded) (Table S1). Normal distribution of nuclear abnormalities was tested using the D’Agostino and Pearson test and statistical testing compared the *LMNA* mutant group (N = 6; 3 cell populations in 2 experiments) versus the non-mutant group (N = 6; 3 cell populations, 2 experiments) using Mann-Whitney U test, because one cell population per group did not have a normal distribution of values.

### Ethics

Work with human cells was performed under approval of the National Health Service (NHS) Health Research Authority Research Ethics Committee (reference No. 13/LO/1826) and Integrated Research Application System (IRAS) project (ID No. 141100) and, for motor neuron work, according to approved regulations and guidelines by University College London Hospital’s National Hospital for Neurology and Neurosurgery and University College London’s (UCL) Institute of Neurology joint research ethics committee (09/0272). Use of hESCs was approved by the Steering Committee for the UK Stem Cell Bank and for the use of stem cell lines (SCSC12-46 and SCSC13-14). Procedures involving animals were approved by the UK Home Office according to Animals (Scientific Procedures) Act (ASPA) regulations and performed under PPL 70/8566.

### Statistics

Values are mean ± SD or SEM (as specified). Specific N or n values and statistical tests are indicated in figure legends. Data were analyzed with Microsoft Excel and GraphPad Prism.

## Data and Software Availability

The raw data reported in this paper have been deposited in Mendeley Data and are available at https://doi.org/10.17632/d826fxhr3b.1.

## Supplemental Information

Document S1. Supplemental Experimental Procedures, Figures S1–S3, and Table S1

Video S1. Nuclear Morphology in WT Artificial Muscles, Related to Figure 3 3D reconstruction of nuclear morphology (LAMIN A/C in green) in a human artificial muscle derived from healthy donor hiPSC (WT control).

Video S2. Nuclear Morphology in *LMNA* Mutant Artificial Muscles, Related to Figure 3 3D reconstruction of nuclear morphology (LAMIN A/C in green) in a human artificial muscle derived from LMNA-mutant hiPSC. Note the presence of elongated and deformed nuclei.

Document S2. Article plus Supplemental Information
