# Supplementary material for: Three-Dimensional Human iPSC-Derived Artificial Skeletal Muscles Model Muscular Dystrophies and Enable Multilineage Tissue Engineering
Source: Cell Rep. 2018 Apr 17;23(3):899–908. doi: 10.1016/j.celrep.2018.03.091 (PMC5917451; doi:10.1016/j.celrep.2018.03.091)
Supplement: Document S1. Supplemental Experimental Procedures, Figures S1–S3, and Table S1 [file mmc1.pdf]

Cell Reports, Volume *23*

Supplemental Information

Three-Dimensional Human iPSC-Derived Artificial Skeletal Muscles Model Muscular Dystrophies and Enable Multilineage Tissue Engineering

Sara Martina Maffioletti, Shilpita Sarcar, Alexander B.H. Henderson, Ingra Mannhardt, Luca Pinton, Louise Anne Moyle, Heather Steele-Stallard, Ornella Cappellari, Kim E. Wells, Giulia Ferrari, Jamie S. Mitchell, Giulia E. Tyzack, Vassilios N. Kotiadis, Moustafa Khedr, Martina Ragazzi, Weixin Wang, Michael R. Duchen, Rickie Patani, Peter S. Zammit, Dominic J. Wells, Thomas Eschenhagen, and Francesco Saverio Tedesco

SUPPLEMENTAL FIGURES, TABLE AND VIDEOS

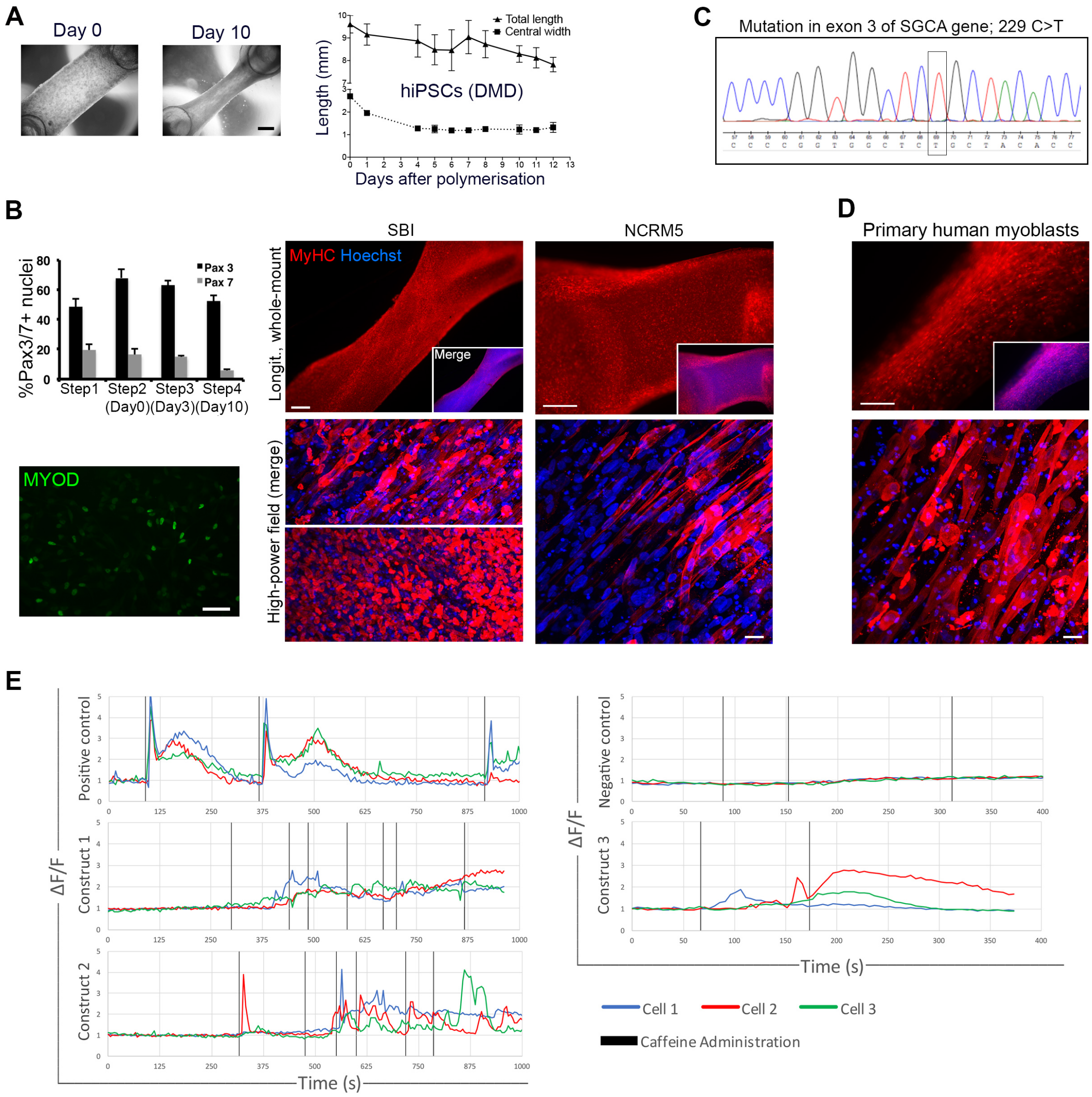

Figure S1. Additional characterization of hPSC-derived 3D artificial muscle constructs, related to Figure 2. (A) Representative phase contrast images of the morphology of cellularized hydrogels from a dystrophic patient (DMD) after polymerization (day 0) and after 10 days in culture. Gels undergo remodeling over time, as also shown by the graphs displaying shortening and thinning in culture. Data plotted as mean ± SD, N = 2 to 4 / point / line. **(B)** Generation of 3D muscle constructs from small-molecule based, transgene-free differentiation of hiPSCs as in (Caron et al., 2016). Histograms on the left show the percentage of PAX3^{+} and

PAX7^{+} cells during monolayer differentiation (quantified by immunofluorescence staining). Bottom left immunofluorescence staining shows nuclear MYOD, demonstrating skeletal myogenic commitment the cultured cells. The two vertical immunofluorescence panels show MyHC staining as shown in Figure 1A in two additional WT iPSC lines. Some hiPSC lines (such as SBI) occasionally showed suboptimal alignment of myotubes in some gels, as shown in the bottom left high power field image. **(C)** Sequencing electropherogram of differentiated LGDM2D-iPSCs used for the experiments confirming the pathological point mutation in exon 3 of the alpha sarcoglycan (SGCA) gene. **(D)** Immunofluorescence panel as Figure 2A showing comparable differentiation and myofiber alignment in artificial muscle constructs generated using primary biopsy-derived human myoblasts. **(E)** Traces showing analysis of calcium transients in muscle constructs loaded with the fluorescent Ca^{2+} indicator Fluo-4AM upon caffeine administration. Three examples of representative responding cells per sample are shown. Vertical back lines indicate time of caffeine administration (10 mM). Three representative constructs out of the 4 tested are shown. Positive control: monolayer of human myotubes; negative control: undifferentiated cells (same line used in the constructs). ΔF/F: measured fluorescence relative to the average resting fluorescence of each point, before the administration of caffeine. Image analysis was conducted with ImageJ and Microsoft Excel was used to generate the graphs. Additional details in Supplemental Experimental Procedures. Scale bars: (A) 1mm (C) 100 μm MYOD and 400 μm others; (D) 400 μm.

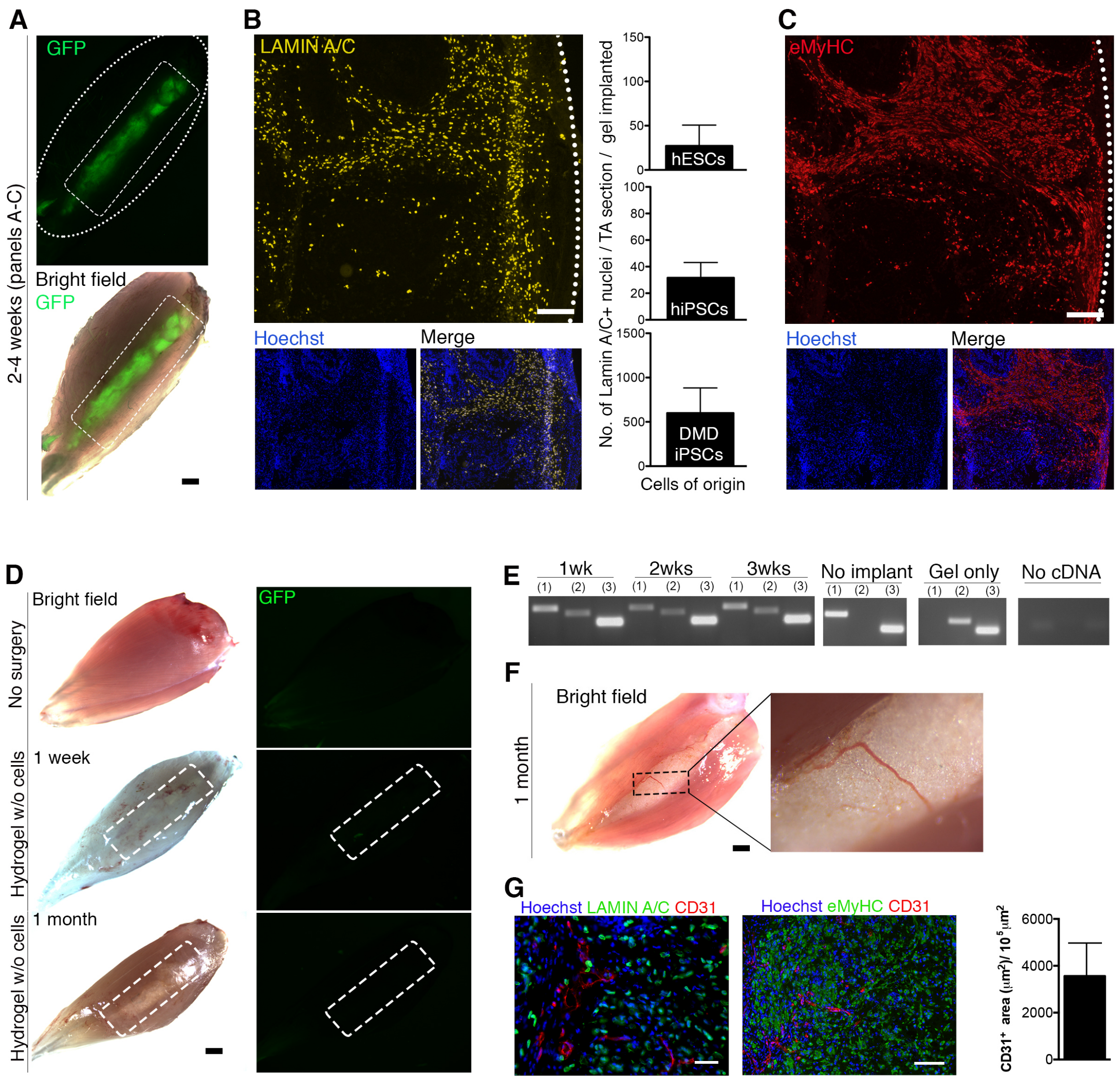

Figure S2. *In vivo* implantation, engraftment and vascularization of hPSC-derived artificial muscles in immunodeficient mice, related to Figure 2.

(A) Representative images of a whole tibialis anterior (TA) muscle 2-4 weeks after implantation in NSG mice. GFP^+ myogenic cells were used to generate artificial muscles. The white dashed squares and ovals indicate the artificial muscle and the mouse TA respectively. **(B)** Immunofluorescence images highlight the presence of engrafted human nuclei (LAMIN A/C^+) in transverse sections of TA muscles 2-4 weeks after implantation Data are from three different healthy or dystrophic cell lines; mean ± SD per TA section (normalized per implanted hydrogel): hESCs 27.14 ± 23.53; hiPSCs 31.59 ± 11.50; DMD hiPSCs 599.13 ± 284.06). Bar graphs on the right show quantification of the number of human nuclei in each muscle section. Bars

show mean ± SD; N = 7 for DMD, 3 for hiPSCs and 5 for hESCs. **(C)** Immunofluorescence staining for embryonic MyHC (eMyHC) of a serial section of the muscle shown in (B). **(D)** Images of whole TA muscles 1 week and 1 month after implantation of hydrogels without cells (middle and bottom images). A control muscle (no surgery) is shown at the top. The hydrogels do not display any autofluorescence in the GFP channel. **(E)** RT-PCR showing expression of alpha sarcoglycan (SGCA; 2) in the transplanted mouse TA at different time points. Control muscles only express mouse Sgca (1) whereas human artificial muscles cultured *in vitro* only express human SGCA. GAPDH was used as loading control (3). **(F)** Bright field images of a freshly explanted TA muscle 1 month after implantation showing blood vessels crossing the implant. **(G)** Immunofluorescence staining of human nuclei (lamin A/C), newly generated myofibres (eMyHC) and endothelial cells (CD31) within the transplanted artificial muscle in TA transverse sections 1-2 weeks after surgery. The area of $CD31^+$ structures present within the implant is quantified in the histogram on the right, showing the mean area normalized per 10^5 μm^2. 300 $CD31^+$ structures in total were measured in 5 pictures. Error bars: SD. Scale bars: (A,D,F) 1.5 mm; (B,C) 200 µm; (G) 50 µm left and 100 µm right.

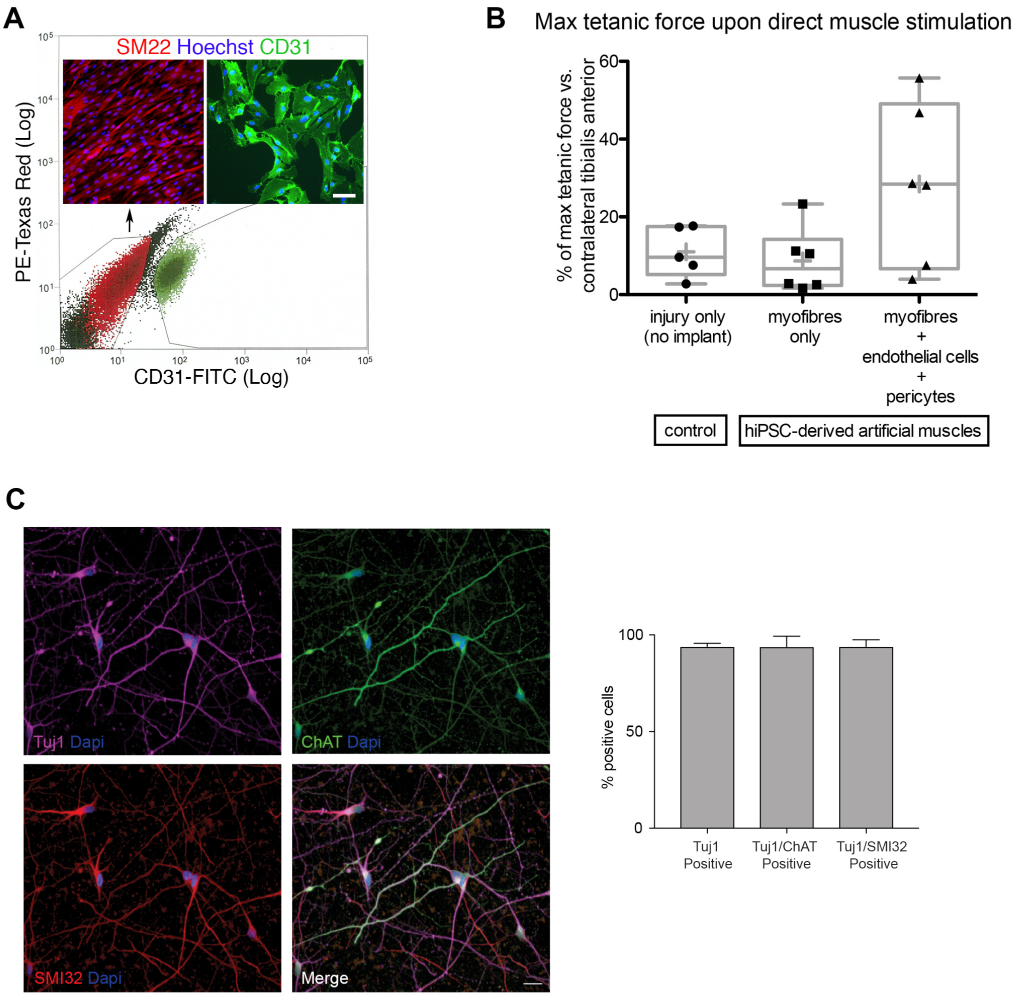

Figure S3. Multilineage hiPSC-derived skeletal muscle constructs: purification of vascular cells, assessment of muscle function *in vivo* and characterization of motor neurons, related to Figure 4. (A) FACS plot depicting isolation of CD31^{+} endothelial cells (ECs) and CD31^{-} pericytes (PCs; immunolabeled also with SM22) derived from isogenic iPSCs used for muscle generation. Details in Experimental Procedures. **(B)** Scatter plot graph (black symbols) with superimposed box-and-whiskers (gray) showing percentage of maximum tetanic force of tibialis anterior muscles (relative to uninjured contralateral muscles) of NSG mice implanted with hiPSC-derived artificial muscles (containing only myofibres or multicellular, i.e. myofibres + ECs + PCs; control: injury and no implant). One-way ANOVA, P = 0.0521; N = 17 mice (corresponding to each dot in the graph). Kruskal-Wallis test was also performed taking into account the N values per group and showed a P value of 0.127. Boxes: 25th to 75th percentiles; horizontal lines: median; “+” symbol: mean; whiskers: min to max values. **(C)** Efficiency of motor neuron (MN) generation

from hiPSCs assessed by immunofluorescence. Cultures were stained for the pan-neuronal marker Tuj1 (pink) and two MN-specific markers: choline acetyltransferase (ChAT) and SMI32. Data representative of differentiation from four different hiPSC lines, of which one was used in 3D co-culture experiments (ThermoFisher A18945). Nuclei were counterstained with Dapi (blue). Scale bars: 20 µm. Error bars: SD.

OPERATOR 1

	Elongated Nuclei	Nuclei with Blebs	Deformed Nuclei	Abnormal Nuclei	Normal Nuclei	Total Number of Nuclei
Control 1 (WT hiPSCs)	5.5% (8)	0% (0)	0.7% (1)	6.2% (9)	93.8% (137)	146
Control 2 (LGMD2D)	1.1% (1)	0% (0)	4.3% (4)	5.4% (5)	94.6% (88)	93
Control 3 (hESCs)	3.9% (4)	0% (0)	6.9% (7)	10.8% (11)	89.2% (91)	102
K32del	5.3% (5)	0% (0)	12.8% (12)	18.1% (17)	81.9% (77)	94
L35P	40.2% (39)	4.1% (4)	8.3% (8)	52.6% (51)	47.4% (46)	97
R249W	23.2% (36)	0% (0)	11% (17)	34.2% (53)	65.8% (102)	155

OPERATOR 2

	Elongated Nuclei	Nuclei with Blebs	Deformed Nuclei	Abnormal Nuclei	Normal Nuclei	Total Number of Nuclei
Control 1 (WT hiPSCs)	8.1 % (16)	1% (2)	0% (0)	9.1% (18)	90.9% (179)	197
Control 2 (LGMD2D)	5.6% (5)	0% (0)	2.3% (2)	7.9% (7)	92.1% (82)	89
Control 3 (hESCs)	4.4% (5)	0% (0)	7.9% (9)	12.3% (14)	87.7% (100)	114
K32del	20.9% (23)	0% (0)	0.9% (1)	21.8% (24)	78.2% (86)	110
L35P	46.6% (48)	4.9% (5)	0.9% (1)	52.4% (54)	47.6% (49)	103
R249W	28.8% (59)	0% (0)	2.9% (6)	31.7% (65)	68.3% (140)	205

OPERATOR 3 (BLINDED)

	Elongated Nuclei	Nuclei with Blebs	Deformed Nuclei	Abnormal Nuclei	Normal Nuclei	Total Number of Nuclei
Control 1 (WT hiPSCs)	4.8% (8)	1.2% (2)	0% (0)	6% (10)	94% (158)	168
Control 2 (LGMD2D)	4.2% (4)	2.1% (2)	3.1% (3)	9.4% (9)	90.6% (87)	96
Control 3 (hESCs)	2.4% (3)	1.6% (2)	1.6% (2)	5.6% (7)	94.4% (117)	124
K32del	17.4% (17)	2% (2)	3.1% (3)	22.5% (22)	77.6% (76)	98
L35P	40% (42)	4.8% (5)	1.9% (2)	46.7% (49)	53.3% (56)	105
R249W	28.4% (50)	0% (0)	1.7% (3)	30.1% (53)	69.9% (123)	176

Table S1. Scoring of nuclear abnormalities in 3D reconstructed LMNA-mutant artificial muscles, related to Figure 3 and Experimental Procedures. The three sub-tables show consistency of scoring nuclear abnormalities across different independent operators. Absolute values shown in brackets.

SUPPLEMENTAL EXPERIMENTAL PROCEDURES

Cell cultures and differentiation protocols

15 distinct hPSC lines (1 hESC + 15 hiPSC lines) and 1 primary human myoblast line were utilized in this study. Both transgene-based and transgene-free skeletal myogenic differentiation protocols of hPSCs were utilized in this work. hPSC-derived inducible myogenic cells (transgene-based; also known as HIDEMs/HEDEMs) were derived and maintained in culture as previously described (Maffioletti et al., 2015; Tedesco et al., 2012). Shef-6 cells (hpscreg.eu/cell-line/UOSe006-A) were used for hESC experiments and were kindly provided by Prof. P. Andrews (University of Sheffield, UK). The following 5 healthy donor hiPSC lines were used: 1) SBI iPSCs (System Biosciences SC102A-1N; hpscreg.eu/cell-line/SBIi006-A; kindly provided by Prof. VandenDriessche, VUB, Belgium)(Maffioletti et al., 2015); 2) in-house reprogrammed healthy control line no.4 (Tedesco et al., 2012); 3) Gibco® Episomal hiPSC Line A13777, LifeTechnologies; 4) NCRM1 (hpscreg.eu/cell-line/CRMi003-A); 5) NCRM5 (hpscreg.eu/cell-line/CRMi001-A). The following five muscular dystrophy hiPSC lines were used: 1) DMD iPSCs (deletion of exons 4-43)(Kazuki et al., 2010) were kindly provided by Prof. M. Oshimura (Tottori University, Japan); 2) LGMD2D iPSC were generated from somatic cells provided by the Munich Tissue Culture Collection (MTCC; Friedrich-Baur Institute, Munich, Germany), were previously described (Tedesco et al., 2012) and are also deposited in hpscreg.eu (UCLi005-A); 3) Three distinct *LMNA*-mutant iPSC lines were reprogrammed and provided by Cellular Dynamics International Inc. and CureCMD. Inducible myogenic cells were differentiated in DMEM, 2 % horse serum, 2 mM glutamine, 100 IU + 0.1 mg / ml penicillin/streptomycin (i.e. differentiation medium) on Matrigel-coated dishes; 1 µM 4-OH tamoxifen (Sigma, H7904) was added twice to the medium, once in proliferation medium when cells reached 90%-100% confluence and the following day in differentiation medium as previously described (Maffioletti et al., 2015; Tedesco et al., 2012). For transgene-free myogenic differentiation, 3 healthy hiPSC lines (NCRM1, NCRM5 and A13777, details above) were differentiated using a published and commercially available small molecule-based protocol (Caron et al., 2016)(Genea Biocells, Skeletal Muscle Differentiation Kit, manufacturer's instructions were followed with minor adaptations). Primary human skeletal myoblasts were

purchased from ThermoFisher (GIBCO® Human Skeletal Myoblasts, cat. no. A12555; grown and differentiated following manufacturer's instructions).

Endothelial cells and pericytes were derived from SBI iPSCs by adapting a previously published protocol (Orlova et al., 2014). Specifically, Gentle Cell Dissociation Reagent was used to create small hiPSC aggregates (STEMCELL Technologies, 07174), which were plated on Matrigel (according to (Orlova et al., 2014)) or Vitronectin (Stemcell Technologies, 07180). Cells were FACS-sorted into $CD31^+$ and $CD31^-$ fractions, obtaining comparable results to the Dynabead-based method described in the original paper. Endothelial cells were cultured as previously published (Orlova et al., 2014), whereas pericytes were cultured as described in Orlova et al. for the initial passages and then adapted to a previously described MegaCell (Sigma)-based medium optimized for skeletal muscle pericytes (Maffioletti et al., 2015; Tedesco et al., 2012) to improve expansion.

Motor neurons were differentiated from 5 hiPSC lines as recently reported (Hall et al., 2017; Stacpoole et al., 2011): a) System Biosciences SC102A-1N; b) Thermo Fisher A18945; c) Coriell ND41866*C and two in-house reprogrammed healthy control lines (Patani Lab). Derivatives from System Biosciences SC102A-1N and Thermo Fisher A18945 hiPSCs where then used in 3D co-culture experiments.

Generation of 3D artificial muscle constructs (extended version)

Fibrin gels were produced as previously published (Hansen et al., 2010) using 3.5 mg/ml human fibrinogen (TISSUCOL DUO 500, Baxter) and 10% Matrigel (BD, 356230) polymerized by adding 3 U/ml thrombin (Biopur, 10-13-1104) to the solution. The silicone posts used for gel polymerization are commercially available (EHT Technologies, GmbH Hamburg). 1×10^6 cells were used for each artificial muscle (total volume 120µl). Artificial muscles were kept in culture at 37°C with 5% CO_2 supplementing the medium with 33 µg / µl aprotinin (Sigma, A3428) to prevent fibrinogen degradation. To induce myogenic differentiation, 1 µM 4-OH tamoxifen was added 48 hours after polymerization in proliferation medium and then 24 hours later in differentiation medium. Multi-lineage artificial muscles were generated with the same method using a cell mix composed of 70% myogenic cells (7×10^5) and 30% vascular cells (6×10^4 ECs and 2.4×10^5 PCs; ratio of 1 EC : 4 PCs). Artificial muscles were cultured in a 1:1 mix of HIDEMs proliferation medium and endothelial medium A (EC-SFM basal medium, 1% Platelet-poor plasma-derived serum, 30

ng/ml VEGF, 20 ng/ml bFGF) for 48 hours then in a 1:1 mix of HIDEMs differentiation medium and endothelial medium B (Lonza, CC-3162) after the second 4-OH Tamoxifen administration. Artificial muscles were kept in culture for 10 days at 37°C with 5% CO_2, changing the medium every other day. Human fibrinogen was kindly provided by Prof. H. Redl (LBG, Vienna).

Immunostaining

For 2D immunofluorescence staining (on cells and muscle sections), samples were fixed in 4% paraformaldehyde (PFA) for 10 minutes at room temperature (RT), washed twice in phosphate buffered saline (PBS) and incubated for 30 minutes at RT with PBS - 1% bovine serum albumin (BSA) - 0.2% Triton. Samples were then incubated with 10% donkey serum for 30 minutes at RT (blocking solution) and then with the primary antibody for either 1 hour at RT or overnight (O/N) at 4°C. Unbound antibody was washed with PBS - 0.2% Triton and the cells were incubated with the secondary antibody and Hoechst 33342 for 1 hour at RT. Samples were imaged using an inverted fluorescence microscope. Artificial muscles were fixed ON in 4% PFA at 4°C, incubated for 6 hours in TBS 1X pH 7.4, 10 % FBS, 1 % BSA, 0.5% Triton X-100 at 4 °C and then with the primary antibodies in TBS 1X pH 7.4, 1 % BSA, 0.5 % Triton X-100 at 4 °C O/N. Samples were then washed over day in TBS 1X at RT and the secondary antibodies incubated in TBS 1X pH 7.4, 1 % BSA, 0.5 % Triton X-100 at 4 °C O/N. Samples were then rinsed again over day in TBS 1X at RT and subsequently imaged. For imaging, whole gels were placed on glass slides with an indentation (Carl Roth, H884.1) in fluorescence mounting medium (Dako, S3023) under a coverslip and imaged using a confocal microscope or an upright fluorescence microscope. For sectioning, fixed gels were dehydrated with a gradient of sucrose (from 7.5 % to 30 %) and then included in Tissue-Tek® O.C.T™ (4583, Sakura) for cryostat processing (10 µm thick slices). Sections were then stained as detailed for 2D cell staining and imaged using an upright fluorescence microscope (Leica). Primary antibodies used: mouse anti-myosin heavy chain (MyHC; MF20, DSHB), rabbit anti-myosin (Calbiochem, 476126), chicken anti-laminin (Abcam, ab14055), mouse anti-Lamin A/C (Novocastra, NCL-LAM-A/C), CD31 (BD, 550274). The secondary antibodies were donkey anti-mouse, goat anti-chicken, donkey anti-

rabbit (all Molecular Probes, Alexa Fluor series) used in combination with Hoechst 33342 (Fluka, B2261).

For immunohistochemistry, PFA-fixed artificial tissues were dehydrated and embedded in paraffin. Longitudinal or transverse paraffin sections (4 μm) were subjected to standard hematoxylin and eosin staining or immunohistochemistry with anti-mouse immunoperoxidase polymer (Histofine Simple Stain MAX PO, Nichirei Biosciences) and universal DAB detection kit (Ventana) after incubation with primary antibody (sarcomeric actin, Dako, M0874).

For the quantification of $CD31^+$ structures within the artificial muscle implant, tibialis anterior sections processed and stained for CD31 (to highlight endothelial cells/vessels), human LAMIN A/C (to highlight the implant) and Hoechst as described above. The area of $CD31^+$ structures within the artificial muscle was calculated with Image J, excluding the area of their lumen when present. The implant area was also calculated for each single muscle section. Data normalized to a field of 1×10^5 μm^2.

DNA extraction and sequencing

QIAamp DNA Mini Kit (Qiagen, 51304) was used to extract DNA from cell pellets following manufacturer's instructions. Sequencing was performed by Source BioScience (Nottingham, UK) using the following primers: GAATCCCCTCTCCTCGCTTC (F) and GATCTTCTGGGGTGGCAGAG (R).

RNA extraction and expression analyses (RT-PCR and qRT-PCR)

RNA was extracted from cell pellets using the RNeasy Mini kit (Qiagen, 74104) following manufacturer's instructions. Trizol reagent (Life technologies, 15596-026) was used to extract RNA from artificial muscles after homogenization as detailed in the manufacturer's protocol. RNA yield and purity was assessed with a Nanodrop; variable amounts of RNA (between 500 ng and 1 μg) were then retro-trascribed to cDNA with the ImProm-II™ Reverse Transcription System kit (Promega, A3800). A PCR for the housekeeping gene GADPH was performed in order to evaluate the efficacy of the retro-transcription reaction. Quantitative Real Time-PCR to assess for the expression of myogenic factors was performed with the GoTaq® qPCR Master Mix kit (Promega, A6001) following manufacturer's directions using a BioRad CFX96 machine. For each experiment, samples were analyzed with technical duplicates/triplicates; N = 3 for all lines apart from *LMNA*-mutant and LGMD2D

hiPSCs, whose error bars in Figure 2E represent intra-experimental replicates (n = 3). Ct data were normalized using GADPH gene expression values for each sample analyzed using the ΔΔCt method (Schmittgen and Livak, 2008) and presented as mean +/- standard deviation. Please find below a list of primers used for expression analyses:

Gene	Primer sequence	Annealing Temperature
GADPH (F)	AGGTCGGTGTGAACGGATTTG	60 °C
GADPH (R)	TGTAGACCATGTAGTTGAGGTCA	60 °C
MYOD (F)	CACTCAAGCGCTGCACGTCG	64 °C
MYOD (R)	GGCCGCTGTAGTCCATCATGC	64 °C
MyHC (F)	GCATCGAGCTCATCGAGAAG	60 °C
MyHC (R)	CATACAGCTTGTTCTTGAAGGAGG	60 °C
Sgca (F; mouse)	GCCCTGCCAAGATGGGTGCTC	60 °C
Sgca (R; mouse)	GCCTTTGTGGAGCCGGAGGTC	60 °C
SGCA (F; human)	GCCTCCACTTCTGTCTTGCT	64 °C
SGCA (R; human)	CCACCAAGAAGTCACGGTCT	64 °C
DYSTROPHIN (F)	GCAAGAGCAACAAAGTGGCCTA	60 °C
DYSTROPHIN (R)	AGCTTCTTCCAGCGTCCCTCA	60 °C

GADPH: glyceraldehyde 3-phosphate dehydrogenase; MYOD: myogenic differentiation 1; MyHC: myosin heavy chain; SGCA: alpha sarcoglycan; F: forward; R: reverse.

Western blot

Proteins were extracted with cold lysis buffer (50 mM Tris HCL pH 7.4, 1 % Triton X-100, 1 mM EDTA pH 8, 150 mM NaCl in ddH_2O) with 1 mM PMSF (Thermo Scientific, 36978) and a protease inhibitor cocktail (Roche, 04693159001). Samples were homogenized on ice. Samples were incubated on ice for 30 minutes and centrifuged at 10000 rpm for 10 minutes at 4°C. Sample concentration was determined using a colorimetric reaction (BioRad, 500-0113). 30µg of proteins per sample were loaded in reducing loading buffer (Alpha Aesar, J61337) on a precast gel (4-15%; Biorad, 345-0028) and run at 100 V. Proteins were transferred onto a PVDF membrane, which was then blocked with 5% non-fat dry milk (Cell Signaling,

999S) in TBST and incubated ON at 4°C with the primary antibody. The membrane was washed three times with TBST, incubated with the secondary antibody for 1 hour at RT and rinsed with TBST. The blot signal was developed with ECL reagents (GE Heathcare, RPN2209) and imaged using a chemi-luminescence detector (Biorad, ChemiDoc MP). Anti-beta-tubulin antibody was used as loading control. The antibodies used were the following: mouse anti-MyHC (DSHB, MF20), mouse anti beta-Tubulin (Sigma, T4026), Anti-Mouse IgG (H+L)-HRP Conjugate (Biorad, 1706516).

Electron microscopy

Samples were washed twice in PBS and incubated for 10 minutes at 37 °C in 30 nM 2,3-butanedione monoxime (Sigma, cat. no. B0753). Samples were then fixed O/N at 4 °C in 0.36 % glutaraldehyde (Sigma, cat. no. G7526). Post-fixation was performed with 1 % osmium tetroxide (Science Services, cat. no. E19110) for 2 hours. Samples were then dehydrated, embedded into a glycid ether-based resin and processed in 50 nm-thick sections. Images were acquired on a Zeiss LEO 912AB microscope.

Imaging and analysis of calcium transients

Calcium transients were measured after loading hiPSC-derived artificial muscle constructs with the fluorescent Ca^{2+} indicator Fluo-4AM (ThermoFisher). A monolayer of differentiated human myotubes was used as a technical positive control for reagents and setup, whereas undifferentiated cells (same line used in the constructs) was used as negative control. Fluorescent dye loading and image acquisition was conducted in Recording Buffer (RB: Glucose 10 mM, NaCl 150 mM, KCl 4.25 mM, NaH_2PO_4 1.25 mM, $NaHCO_3$ 4mM, $CaCl_2$ 1.2 mM, $MgCl_2$ 1.2 mM, HEPES 10 mM, pH 7.4) at 37°C. Cell preparations were loaded in RB containing Fluo-4AM 5 µM and Pluronic 0.002% (w/v) for 30 minutes which was washed off with RB prior to image acquisition on the thermostat stage (at 37°C) of a Zeiss 880 CLSM. Fluorescence was excited at 488 nm, emission collected at >530 nm and images were acquired as a time series. Additions of caffeine in RB at 10 mM concentration were used to stimulate SR Ca^{2+} release via ryanodine receptors (RyR). Muscle constructs often required multiple doses of caffeine and a delayed response was also observed in some cells, possibly due to the layer of fibrin covering the myotubes and delaying interaction of caffeine with the cell. Muscle constructs were removed from

their silicone posts for imaging, which together with the handling before imaging might explain the presence of non-responding cells together with responders. Nonetheless, cells showing caffeine-induced calcium transients were detected in all 3D muscle constructs analyzed (n = 4; 3 examples shown in figure S1). Different regions of the recorded field were tracked to record the fluorescent intensity (corresponding to a small area of one cell, approximately 10% of the cell area, to avoid interfering signals from other cells in 3D). A minimum of 20 total traces was analyzed and three representative responding cells per muscle are shown in Figure S1H. The graph shows ΔF/F traces, which is the measured fluorescence relative to the average resting fluorescence of each point, before the administration of caffeine. Image analysis was conducted with ImageJ and Microsoft Excel was used to generate the graphs.

In vivo **implantation studies**

Adult NOD-scid-gamma immunodeficient mice (NSG) were used for transplantation assays (N = 24). Animals were anesthetized with isoflurane (in O_2 at 2 liters / minute) and the surgery was performed under sterile aseptic technique. Briefly, a longitudinal incision was performed on the skin on top of the tibialis anterior (TA) muscle to expose the muscle tissue. A 2 mm deep, 8 mm longitudinal incision was then performed to open an intramuscular pocket and a volume of muscle tissue corresponding to the hydrogel(s) needing implantation removed. Occasional bleeding was controlled with a high temperature cauterizer (Bovie Medical, AA01). Before implantation, gels were washed twice with sterile PBS and then implanted into the muscle pocket. 8μL of fibrin glue (TISSUCOL DUO 500, Baxter) was added on top of the pocket containing the implant to seal the wound and keep the artificial tissue in place. 1 or 2 gels were implanted respectively for short (1 week) or long time (2-4 weeks) analyses. The skin was sutured with 4.0 Vicryl sutures (Ethicon) and the mouse was allowed a gradual recovery from anaesthesia. Analgesia (Carprofen) was administered 40 minutes before surgery and the day after to minimize discomfort. The surgeon was not aware of the genotype or composition of the implants. All mice recovered promptly without post-operative adverse events.

For functional vascularization assays, DyLight® 594 GSL I-B4 isolectin (Vector Laboratories, DL-1207) was injected into the tail vein of NSG mice 1 week after surgical procedure (100 μg/injection). Animals were humanely killed 20 minutes after

injection, muscles dissected and then cryopreserved for further analyses. Isolectin staining was imaged on muscle sections with a fluorescent microscope (excitation wavelength: 592 nm).

Mouse muscle tissue processing

Tissues were harvested from the animals at different time points and cryopreserved as previously described (Gerli et al., 2014). Muscles were sectioned (thickness: 8 μm) using a cryostat (Leica, CM1850) and collected on polarized glass slides. Samples for RNA extraction were collected during muscle sectioning. Sections were stored at –80°C.

In vivo force assessment

Four weeks after surgery (performed as described in the "*In vivo* implantation studies section" above), muscle function was assessed for the right and left tibialis anterior (TA) muscles. Two hydrogels per NSG tibialis anterior muscle were implanted and chimeric muscles were assessed one month after implant. A total of 17 mice and 34 muscles were assessed (N = 6 muscles containing myofibres-only hiPSC-derived hydrogels, 6 muscles containing multicellular hiPSC-derived hydrogels, 5 injury-only muscles and 34 uninjured contralateral muscles). One injury-only mouse was excluded from the analysis because of technical problems during recording of muscle force. The operating surgeon was blind to the type of implanted hydrogel and muscle physiologists were blind to all groups. The muscle function assessment procedure was adapted from standard protocols (Liu M et al., 2005; Sharp PS et al., 2005) and has been previously described (Foster H et al., 2008). Mice were deeply anesthetized with Hypnorm (VetPharma, Leeds, UK) and Hypnovel (Roche, Welwyn Garden City, UK) as previously described and were carefully monitored throughout the experiment to ensure that there was no reflex response to toe pinch. The distal tendon of the TA muscle was dissected from surrounding tissue and tied with 4.0 braided surgical silk (Interfocus, Cambridge, UK). The sciatic nerve was exposed and superfluous branches axotomized, leaving the TA motor innervation via the common peroneal nerve intact. The mouse was placed on a thermopad (Harvard Apparatus, Edenbridge, UK) to maintain body temperature at 37 °C. The foot was secured to a platform and the ankle and knee immobilized using stainless steel pins. The TA tendon was attached to the lever arm of a 305B dual-mode servomotor transducer

(Aurora Scientific, Aurora, Ontario, Canada) via a custom-made steel s-hook. TA muscle contractions were elicited by stimulating the proximal part of common peroneal nerve via bipolar platinum electrodes, using supramaximal square-wave pulses of 0.02 ms (701A stimulator; Aurora Scientific). Data acquisition and control of the servomotors were conducted using a Lab-View-based DMC program (Dynamic muscle control and Data Acquisition; Aurora Scientific). Optimal muscle length (Lo) was determined by incrementally stretching the muscle using a micromanipulator until the maximum isometric twitch force was achieved. Maximum isometric tetanic force (Po) was determined from the plateau of the force-frequency relationship following a series of stimulations at 10, 30, 40, 50, 80, 100, 120, and 150 Hz. A 1-minute rest period was allowed between each tetanic contraction. For direct muscle stimulation two custom made electrodes were inserted in the mid belly of the muscle and the whole procedure was repeated using 4V stimulation. Muscle length was measured using digital calipers based on well-defined anatomical landmarks near the knee and the ankle. Results presented are the ratio of maximum tetanic force of the treated vs the untreated contralateral limb for direct muscle stimulation only, as the injury model and specific timeline appeared not to give sufficient time for innervation and assessment via sciatic nerve stimulation.

SUPPLEMENTAL REFERENCES

Foster, H, Sharp, PS, Athanasopoulos, T, Trollet, C, Graham, IR, Foster, K et al. (2008). Codon and mRNA sequence optimization of microdystrophin transgenes improves expression and physiological outcome in dystrophic mdx mice following AAV2/8 gene transfer. Mol. Ther. *16*, 1825–1832.

Liu, M, Yue, Y, Harper, SQ, Grange, RW, Chamberlain, JS and Duan, D (2005). Adenoassociated virus-mediated microdystrophin expression protects young mdx muscle from contraction-induced injury. Mol. Ther. *11*, 245–256.

Schmittgen, T.D., and Livak, K.J. (2008). Analyzing real-time PCR data by the comparative C(T) method. Nat. Protoc. *3*, 1101-1108.

Sharp, PS, Dick, JR and Greensmith, L (2005). The effect of peripheral nerve injury on disease progression in the SOD1(G93A) mouse model of amyotrophic lateral sclerosis. Neuroscience *130*, 897–910.
