# Supplementary material for: Three-Dimensional Human iPSC-Derived Artificial Skeletal Muscles Model Muscular Dystrophies and Enable Multilineage Tissue Engineering
Source: Cell Rep. 2018 Apr 17;23(3):899–908. doi: 10.1016/j.celrep.2018.03.091 (PMC5917451; doi:10.1016/j.celrep.2018.03.091)
Supplement: Document S2. Article plus Supplemental Information [file mmc4.pdf]

Cell Reports

Resource

Three-Dimensional Human iPSC-Derived Artificial Skeletal Muscles Model Muscular Dystrophies and Enable Multilineage Tissue Engineering

Graphical Abstract

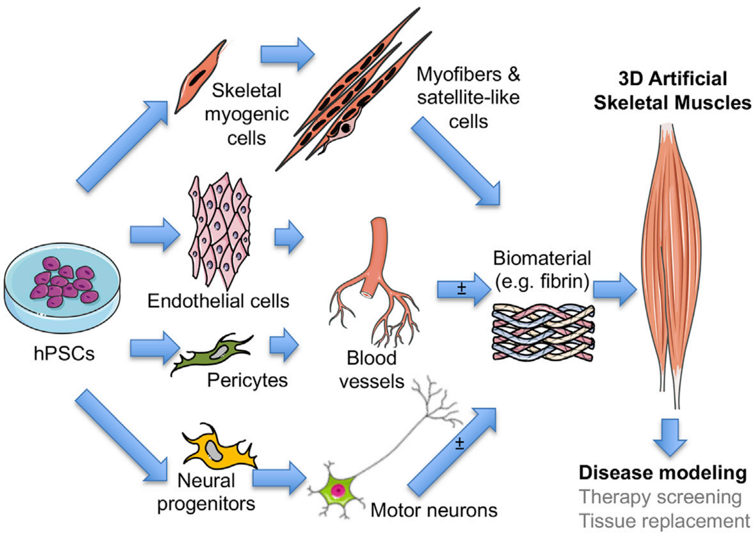

Highlights

- Human iPSC-derived 3D artificial muscles show features of normal skeletal muscle
- Multiple muscular dystrophy iPSC lines can be differentiated in 3D artificial muscles
- Artificial muscle constructs model severe, incurable forms of muscular dystrophy
- Isogenic vascular-like networks and motor neurons develop within artificial muscles

Authors

Sara Martina Maffioletti, Shilpita Sarcar, Alexander B.H. Henderson, ..., Dominic J. Wells, Thomas Eschenhagen, Francesco Saverio Tedesco

Correspondence

f.s.tedesco@ucl.ac.uk

In Brief

Maffioletti et al. generate human 3D artificial skeletal muscles from healthy donors and patient-specific pluripotent stem cells. These human artificial muscles accurately model severe genetic muscle diseases. They can be engineered to include other cell types present in skeletal muscle, such as vascular cells and motor neurons.

Maffioletti et al., 2018, Cell Reports *23*, 899–908
April 17, 2018 © 2018 The Author(s).
https://doi.org/10.1016/j.celrep.2018.03.091

CellPress

Cell Reports
Resource

OPEN ACCESS
CellPress

Three-Dimensional Human iPSC-Derived Artificial Skeletal Muscles Model Muscular Dystrophies and Enable Multilineage Tissue Engineering

Sara Martina Maffioletti,[1,8,9] Shilpita Sarcar,[1,8] Alexander B.H. Henderson,[1] Ingra Mannhardt,[2,3] Luca Pinton,[1,4] Louise Anne Moyle,[1] Heather Steele-Stallard,[1,4] Ornella Cappellari,[5] Kim E. Wells,[5] Giulia Ferrari,[1] Jamie S. Mitchell,[6,7] Giulia E. Tyzack,[6,7] Vassilios N. Kotiadis,[1] Moustafa Khedr,[1] Martina Ragazzi,[1,10] Weixin Wang,[1,11] Michael R. Duchen,[1] Rickie Patani,[6,7] Peter S. Zammit,[4] Dominic J. Wells,[5] Thomas Eschenhagen,[2,3] and Francesco Saverio Tedesco[1,12,*]
[1]Department of Cell and Developmental Biology, University College London, London WC1E 6DE, UK
[2]Department of Experimental Pharmacology and Toxicology, University Medical Center Hamburg Eppendorf (UKE), 20246 Hamburg, Germany
[3]DZHK (German Centre for Cardiovascular Research), partner site Hamburg/Kiel/Lübeck, Germany
[4]Randall Centre for Cell and Molecular Biophysics, King's College London, London SE1 1UL, UK
[5]Department of Comparative Biomedical Sciences, Royal Veterinary College, London NW1 0TU, UK
[6]Institute of Neurology, University College London, London WC1N 3BG, UK
[7]The Francis Crick Institute, London NW1 1AT, UK
[8]These authors contributed equally
[9]Present address: San Raffaele Telethon Institute for Gene Therapy (TIGET), San Raffaele Scientific Institute, 20132 Milan, Italy
[10]Present address: MolMed S.p.A., Milan, Italy
[11]Present address: University College London Institute of Ophthalmology, London EC1V 9EL, UK
[12]Lead Contact
*Correspondence: f.s.tedesco@ucl.ac.uk
https://doi.org/10.1016/j.celrep.2018.03.091

SUMMARY

Generating human skeletal muscle models is instrumental for investigating muscle pathology and therapy. Here, we report the generation of three-dimensional (3D) artificial skeletal muscle tissue from human pluripotent stem cells, including induced pluripotent stem cells (iPSCs) from patients with Duchenne, limb-girdle, and congenital muscular dystrophies. 3D skeletal myogenic differentiation of pluripotent cells was induced within hydrogels under tension to provide myofiber alignment. Artificial muscles recapitulated characteristics of human skeletal muscle tissue and could be implanted into immunodeficient mice. Pathological cellular hallmarks of incurable forms of severe muscular dystrophy could be modeled with high fidelity using this 3D platform. Finally, we show generation of fully human iPSC-derived, complex, multilineage muscle models containing key isogenic cellular constituents of skeletal muscle, including vascular endothelial cells, pericytes, and motor neurons. These results lay the foundation for a human skeletal muscle organoid-like platform for disease modeling, regenerative medicine, and therapy development.

INTRODUCTION

Skeletal muscle is the most abundant human tissue, and it is responsible for movement, posture, temperature control, and various metabolic functions. It is composed of aligned multinucleated myofibers, and its repair and regeneration rely on resident stem or progenitor cells, of which satellite cells are the best characterized (Tedesco et al., 2010). Nonetheless, impaired muscle regeneration occurs in acute or chronic conditions, such as significant trauma (Grogan et al., 2011), or incurable inherited disorders, such as muscular dystrophies (Mercuri and Muntoni, 2013). Artificial human skeletal muscles would provide an invaluable tool to study pathological mechanisms, test potential therapeutics, and develop tissue replacement protocols. Use of a similar approach in other tissues has proved transformational for drug development and regenerative medicine by means of organoid technology (Fatehullah et al., 2016; Lancaster and Knoblich, 2014).

Although several studies have reported methods to engineer rodent skeletal muscle tissue (Carosio et al., 2013; Corona et al., 2014; Huang et al., 2005; Juhas et al., 2014; Machingal et al., 2011; Shandalov et al., 2014; VanDusen et al., 2014), fewer groups have used human cells (Chiron et al., 2012; Fuoco et al., 2015; Madden et al., 2015; Powell et al., 1999; Quarta et al., 2017; Tchao et al., 2013). Apart from a recent study on healthy donor human pluripotent stem cells (hPSCs) for muscle tissue engineering (Rao et al., 2018), most studies used primary human cells from invasive muscle biopsies, facing hurdles such as poor cell availability, limited cell expansion potential, and exhaustion of differentiation ability. In addition, although there is an increasing need to develop clinically relevant multilineage patient-specific models (Giacomelli et al., 2017), no such isogenic human skeletal muscle model has been derived to date. These obstacles hinder the translational potential of these platforms for muscle diseases.

To overcome these limitations, here we have exploited the virtually unlimited proliferative capacity and controllable differentiation of human embryonic stem cells (hESCs) and human

This is an open access article under the CC BY license (http://creativecommons.org/licenses/by/4.0/).

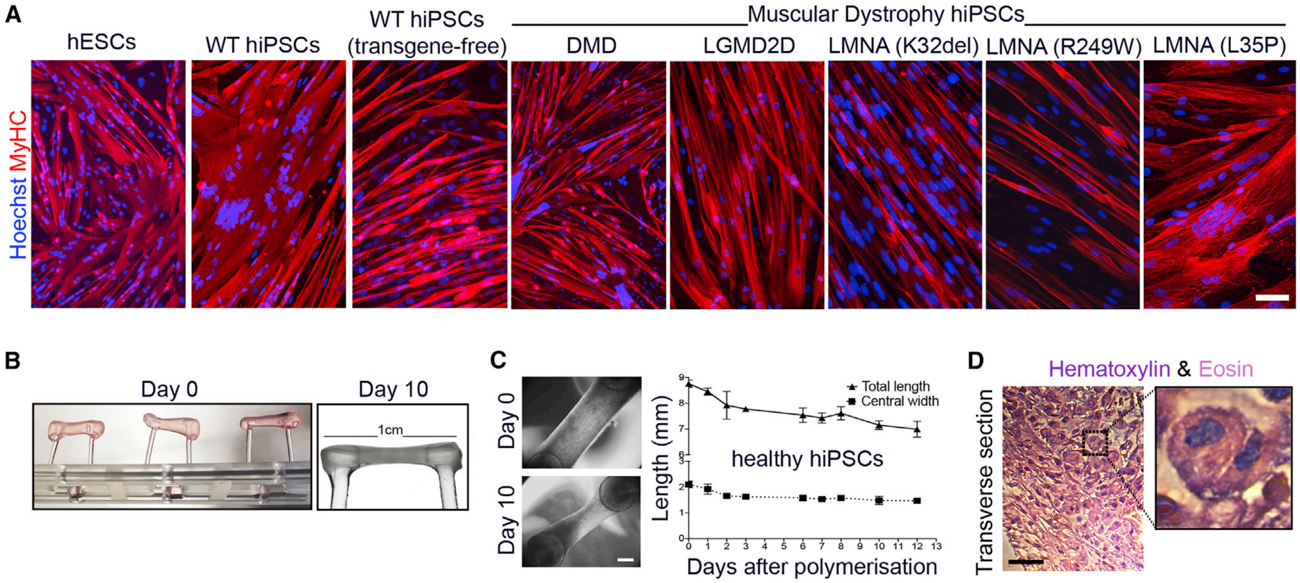

Figure 1. Differentiation of Multiple hPSC Lines into Skeletal Myotubes and Remodeling of Fibrin Hydrogels upon 3D Culture

(A) Immunofluorescence analysis for myosin heavy chain (MyHC) in standard monolayer cultures of differentiated hESC- and hiPSC-derived cells (DMD, Duchenne muscular dystrophy; LGMD2D, limb-girdle muscular dystrophy type 2D; *LMNA*, skeletal muscle laminopathies, specific mutations listed).

(B) Side-view of the 3D culture platform with freshly polymerized gels at day 0 (left) and day 10 (right) of culture containing hiPSC-derived myogenic cells.

(C) Representative phase contrast images of cellularized hydrogels after polymerization (day 0) and after 10 days in culture. Gels undergo remodeling, shorten, and thin in culture (graph). Mean ± SD, N = 2–4 per time point.

(D) H&E staining of a transverse hydrogel sections after 10 days of differentiation.

Magnification: centronucleated myofibers. Scale bars: (A and D) 100 μm; (C) 1 mm.

induced pluripotent stem cells (hiPSCs) (referred to collectively as hPSCs) (Inoue et al., 2014) to produce 3D artificial skeletal muscle constructs for complex muscle disease modeling. hPSCs were induced to skeletal myogenesis within a biocompatible hydrogel using established protocols (Caron et al., 2016; Maffioletti et al., 2015; Tedesco et al., 2012). Artificial muscles could be made using hiPSCs from Duchenne, limb-girdle type 2D, and LAMIN A/C (*LMNA*)-related muscular dystrophies. Our 3D platform modeled cellular hallmarks of *LMNA*-related muscular dystrophies with high fidelity. Finally, essential cell types present in muscle tissue, such as vascular cells and motor neurons, were derived from the same hiPSC source to generate isogenic multilineage muscle constructs.

RESULTS

3D Artificial Muscles Can Be Generated from Multiple Healthy and Dystrophic hPSC Lines

To generate 3D human artificial skeletal muscles, hPSC-derived myogenic cells (healthy and dystrophic) (Figure 1A) were embedded in fibrin hydrogels and differentiated, adapting a cardiac tissue engineering platform (Hansen et al., 2010). Fibrin was polymerized from fibrinogen in molds between two flexible silicone posts providing continuous tension to the gel, sufficient to direct orientation of cells along the force axis (Figure 1B). Over 10 days, cells remodeled the matrix, generating a 7–8 mm long strip of tissue containing structures resembling skeletal myofibers (Figures 1C, 1D, and S1A). Both transgene-based (Maffioletti et al., 2015) and transgene-free (Caron et al., 2016) differentiation protocols produced muscle constructs (Figures 2A and S1B). Transgene-based muscle constructs were generally used due to the better alignment of myofibers, easier scalability of cultures, and experimental cost-effectiveness.

Immunolabeling of artificial muscles from hESCs and hiPSCs—healthy donor, Duchenne muscular dystrophy (DMD), limb-girdle muscular dystrophy type 2D (LGMD2D) (Figure S1C), and *LMNA*-related muscular dystrophies—showed homogeneous presence of myosin heavy chain (MyHC)$^+$ multinucleated myotubes oriented along the force axis of the hydrogels (Figures 2A and 2B), as observed with primary human myoblasts within the same platform (Figure S1D). Transverse sections of artificial muscles revealed abundant MyHC$^+$ muscle fibers surrounded by LAMININ$^+$ extracellular matrix (Figure 2C). Western blot analysis confirmed production of MyHC protein (Figure 2D). Healthy and dystrophic muscle constructs also expressed markers of skeletal muscle determination and maturation (e.g., *MYOD* and *DYSTROPHIN*) (Figure 2E). Immunohistochemical staining highlighted proteins associated with skeletal muscle maturation, such as sarcomeric actin (Figure 2F). Electron microscopy revealed that muscle constructs displayed some degree of cytoskeletal organization into sarcomeres, the basic functional unit of striated myofibers (Figure 2G). Functional myotubes within the artificial muscles were detected by caffeine-induced calcium transients (Figure S1E).

Pax7 marks a population of self-renewing myogenic stem cells known as satellite cells (Tedesco et al., 2010). To assess

OPEN ACCESS
CellPress

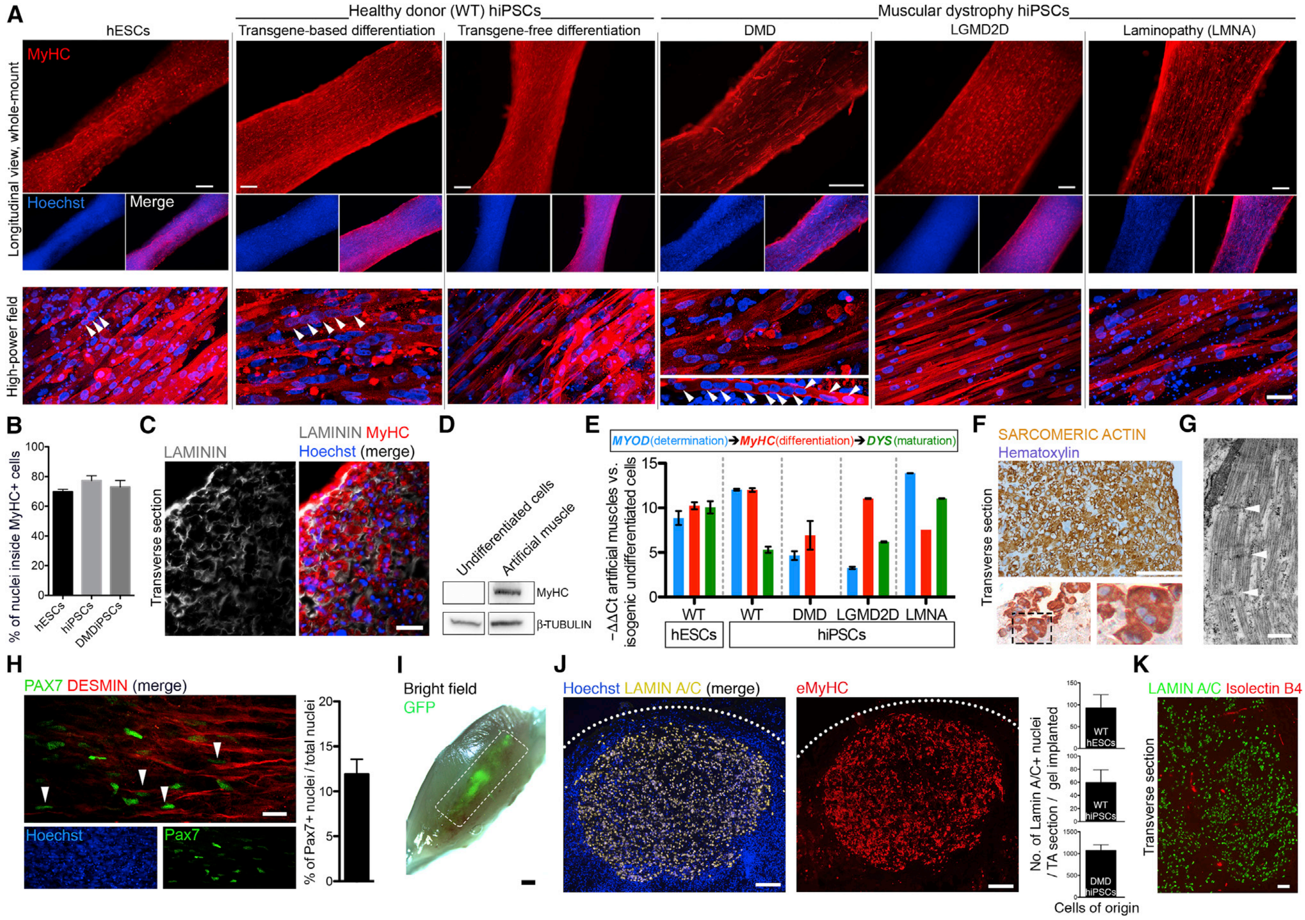

Figure 2. 3D Artificial Skeletal Muscle Constructs Derived from Healthy and Dystrophic hPSCs

(A) Whole-mount immunofluorescence for myosin heavy chain (MyHC) on muscle constructs derived from hESCs, WT hiPSCs (transgene-based and transgene-free differentiation protocols) and dystrophic hiPSCs (DMD, LGMD2D, and skeletal muscle LMNA) differentiated in 3D for 10 days. Nuclei are counterstained with Hoechst. Arrowheads: multinucleated myotubes.

(B) Graph quantifying the proportion of MyHC$^+$ cells using z stack confocal microscopy of three hPSC lines shown in (A).

(C) Immunolabeling for LAMININ (extracellular matrix), MyHC, and nuclei (Hoechst) on DMD artificial muscles.

(D) Western blot for MyHC (250 kDa) in undifferentiated and 3D differentiated iPSC-derived, inducible myogenic cells. β-tubulin: loading control (50 kDa).

(E) qRT-PCR analysis of artificial muscles for myogenic markers. *DYSTROPHIN* (DYS) is absent from DMD-derived artificial muscles. N = 3 for all lines apart from *LMNA* mutant and LGMD2D hiPSCs, whose error bars represent intra-experimental replicates (n = 3). Values are normalized on *GAPDH* expression; ΔΔCt is calculated on the corresponding expression values of undifferentiated cells.

(F) Immunohistochemistry for sarcomeric actin in DMD artificial muscles after 10 days of differentiation.

(G) Transmitted electron microscopy images of DMD iPSC-derived artificial muscle showing sarcomeres (white arrowheads: z lines).

(H) Immunofluorescence showing PAX7$^+$ nuclei adjacent to DESMIN$^+$ myofibers following transgene-free commitment and differentiation of hiPSCs in 3D for 14 days. The graph quantifies the percentage of PAX7$^+$ nuclei within the hydrogels (a total of 5,341 nuclei across 10 random fields).

(I) Bright-field image of a tibialis anterior (TA) muscle 1 week after implantation of artificial muscles generated using GFP$^+$ myogenic cells. Dashed rectangle: grafted area.

(J) Immunofluorescence showing engrafted human nuclei (LAMIN A/C$^+$, left) corresponding to an area in a serial section with embryonic MyHC$^+$ (eMyHC) fibers in transverse sections of a TA muscle 1 week after implantation. Right graphs show quantification of human nuclei from three healthy or dystrophic cell lines; N = 6, 2 mice/cell type; mean ± SD: hESCs 92 ± 30, hiPSCs 59 ± 19, DMD hiPSCs 1,068 ± 132.

(K) Immunofluorescence of systemically delivered 594-conjugated IB4 isolectin (red) labeling endothelial cells within the implanted human artificial muscle (LAMIN A/C: human nuclei).

Error bars: mean ± SD. Scale bars: (A) top 250 μm, bottom 25 μm; (C, F, and K) 100 μm; (G) 1 μm; (H) 20 μm; (I) 1 mm; (J) 200 μm. For additional information, see Figures S1 and S2.

whether our 3D platform generated and provided a niche for Pax7$^+$ cells, we induced myogenic commitment and differentiation of transgene-free hiPSC-derived myogenic progenitors directly in hydrogels. This resulted in PAX7$^+$ cells juxtaposed to myofibers, albeit with variability among the tested hiPSC lines (Figure 2H).

To further characterize hPSC-derived artificial muscles, they were implanted into immunodeficient mice. Tibialis anterior

OPEN ACCESS
CellPress

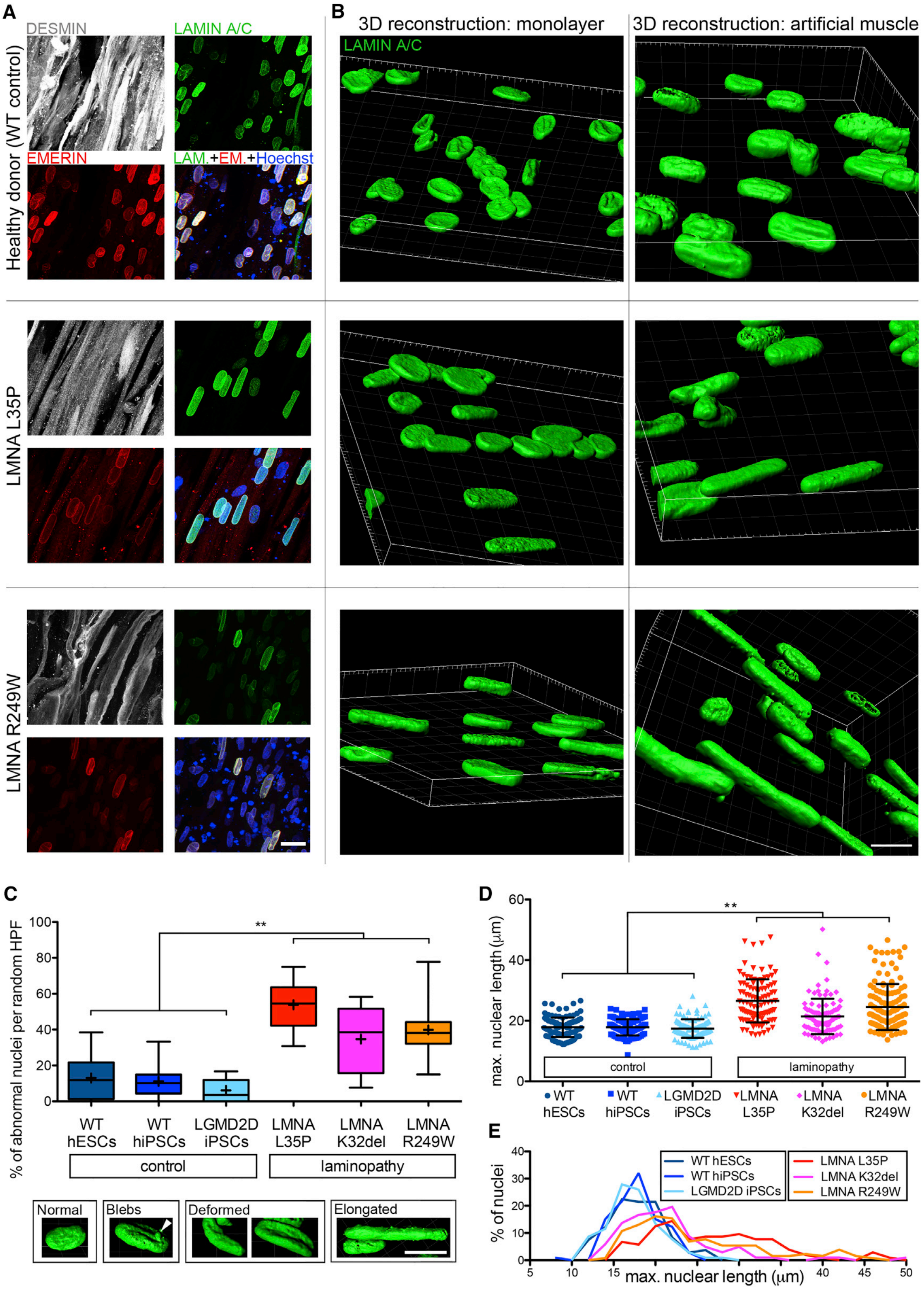

Figure 3. hiPSC-Derived Artificial Skeletal Muscles Model Skeletal Muscle Laminopathies

(A) Confocal (z stacks merge) whole-mount immunofluorescence for DESMIN (myotubes), LAMIN A/C, and EMERIN (nuclear lamina) on hiPSC-derived (healthy and *LMNA* mutant) artificial muscles. Hoechst: nuclei.

(legend continued on next page)

(TA) muscles of non-obese diabetic (NOD)-severe combined immunodeficiency (SCID)-gamma mice (NSG) (N = 24) were injured, and a strip of host tissue was replaced with GFP-expressing hPSC-derived artificial muscles. GFP^{+} implants were identifiable in explanted TA muscles at various time points (Figures 2I and S2A), suggesting successful engraftment. Transverse sections of implanted muscles showed engraftment of human cells, highlighted by the widespread presence of human LAMIN A/C^{+} nuclei (Figures 2J and S2B). LAMIN A/C^{+} areas colocalized with embryonic MyHC^{+} (eMyHC) fibers, demonstrating skeletal muscle generation *in vivo* (Figures 2J, S2C, and S2D). Engraftment was confirmed by expression of human muscle-specific transcripts in implanted muscles, and blood vessels within the implants were detected by immunolabeling for CD31 (Figures S2E–S2G). We further investigated vascularization of the artificial muscle by systemically injecting fluorescent isolectin into the mouse circulation before harvesting implanted muscles. Isolectin^{+} vessels were evident within the implant, confirming functional vascularization (Figure 2K).

Therefore, fibrin hydrogels under uniaxial tension stimulate efficient and aligned 3D skeletal myogenic differentiation of healthy and dystrophic hPSCs. Muscle constructs recapitulate distinctive molecular, structural, and functional features of skeletal muscle and engraft in immunodeficient mice.

hiPSC-Derived Artificial Skeletal Muscles Enable Disease Modeling of Skeletal Muscle Laminopathies

Organoids have great potential for disease modeling and drug development, so we examined whether our organoid-like, artificial skeletal muscle could model severe and incurable forms of muscular dystrophy. We also hypothesized that the 3D nature of our hydrogels would facilitate detection of pathological hallmarks less evident in standard bi-dimensional cultures. To investigate this, we examined artificial muscles generated from hiPSC derived from patients with muscular dystrophies caused by mutations in the *LMNA* gene. *LMNA* mainly encodes the A-type lamins, lamin A and lamin C (LAMIN A/C), nuclear envelope proteins that assemble with B-type lamins into the nuclear lamina, providing structural support and regulating gene expression (Worman, 2012). *LMNA* mutations cause a plethora of diseases called laminopathies, of which three forms affect skeletal muscle (Maggi et al., 2016): limb-girdle muscular dystrophy type 1B (LGMD1B), autosomal dominant Emery-Dreifuss muscular dystrophy 2 (EDMD2), and *LMNA*-related congenital muscular dystrophy (L-CMD). Abnormalities in nuclear morphology are a key histological feature of skeletal muscle laminopathies (Park et al., 2009), and using hiPSC-based modeling could provide a unique, non-invasive tool to address open questions, such as challenging genotype-phenotype correlations, and develop new therapeutics (Scharner et al., 2015).

3D artificial muscles were made by differentiating three *LMNA* mutant hiPSCs from patients with skeletal muscle laminopathies, referred to by their mutation as *LMNA* L35P, R249W, and K32del (Figures 1A, 2A, and 3A). 3D nuclear reconstruction of mutant *LMNA* cells differentiated in artificial muscles highlighted features less prominent in standard monolayer cultures (Figure 3B). This prompted us to quantify nuclear abnormalities, including elongation, deformities, and presence of blebs (Figure 3C), in *LMNA* mutant hiPSC-derived artificial muscles. For controls, we used wild-type (WT) hPSCs and LGMD2D hiPSCs (Figures 1A and 2A), which have not been reported to have dysmorphic nuclei (Kirschner and Lochmüller, 2011). As expected, nuclei in artificial muscle derived from control cells did not display significant nuclear abnormalities. In contrast, all mutant *LMNA* artificial muscles showed a significant proportion of cells with nuclear aberrations ($p = 0.0022$, N = 6) (Figure 3C; Table S1; Videos S1 and S2).

Nuclear elongation was a predominant abnormality, in line with biopsy-derived primary myoblast 3D cultures (Bertrand et al., 2014). Measurements confirmed that laminopathic muscle constructs contained significantly elongated nuclei compared to control artificial muscles ($p = 0.0022$) (Figures 3D and 3E), supporting using this outcome measure in future therapy screening platforms. Thus, hiPSC-derived artificial muscles recapitulate cellular hallmarks of skeletal muscle laminopathies with high fidelity and are amenable to model severe muscle disorders.

Increasing Histological Complexity: Multilineage hiPSC-Derived 3D Artificial Skeletal Muscles

Skeletal muscle tissue also contains non-muscle support cell types, such as vascular endothelial cells (ECs) and pericytes (PCs), which permit blood perfusion while controlling homeostasis of the muscle stem cell compartment (Christov et al., 2007). Therefore, adding ECs and PCs to artificial muscles could generate a more physiologically relevant model *in vitro* and improve survival of larger constructs *in vivo*, which require prompt vascularization to prevent hypoxia-induced cell death (Criswell et al., 2013; Gholobova et al., 2015; Koffler et al., 2011; Levenberg et al., 2005). To achieve these aims, we first derived isogenic ECs and PCs from the same hiPSCs used for myogenic differentiation (Figure S3A) (Orlova et al., 2014). We then combined isogenic hiPSC-derived ECs, PCs, and myogenic

(B) Comparison of confocal 3D nuclear reconstructions of the same iPSC lines shown in (A) differentiated as monolayer cultures (left) versus 3D artificial muscle constructs (right). Nuclei are immunolabeled for LAMIN A/C.

(C) Box and whiskers graph quantifying nuclear abnormalities in hiPSC-derived artificial muscle generated from 3 patients affected by *LMNA*-related muscular dystrophies (red color shades) versus 3 control donors (blue color shades). LGMD2D artificial muscles are included as negative control, because they do not have nuclear abnormalities. Lower panel: representative images of 3D-reconstructed nuclei used to score laminopathy versus control muscles in the graph. **$p = 0.0022$, Mann-Whitney U test. n = 6 in CTRL group and 6 in *LMNA* mutant group (3 cell populations per group in 2 independent experiments). A minimum of 45 nuclei/hydrogel/experiment across 8 random high-power fields were scored. Boxes, 25th to 75th percentiles; horizontal line inside, median; +, mean; whiskers, min to max values.

(D) Scatter dot plot of the specific length of the nuclei scored in (C). **$p = 0.0022$, Mann-Whitney U test. n = 6 in CTRL group and 6 in *LMNA* mutant group (3 cell populations per group in 2 independent experiments). Each symbol is one nucleus. Error bars: mean ± SD.

(E) Distribution plot of the graph in (D).

Scale bars: (A and B) 15 μm; (C) 10 μm. For additional information, see Table S1 and Videos S1 and S2.

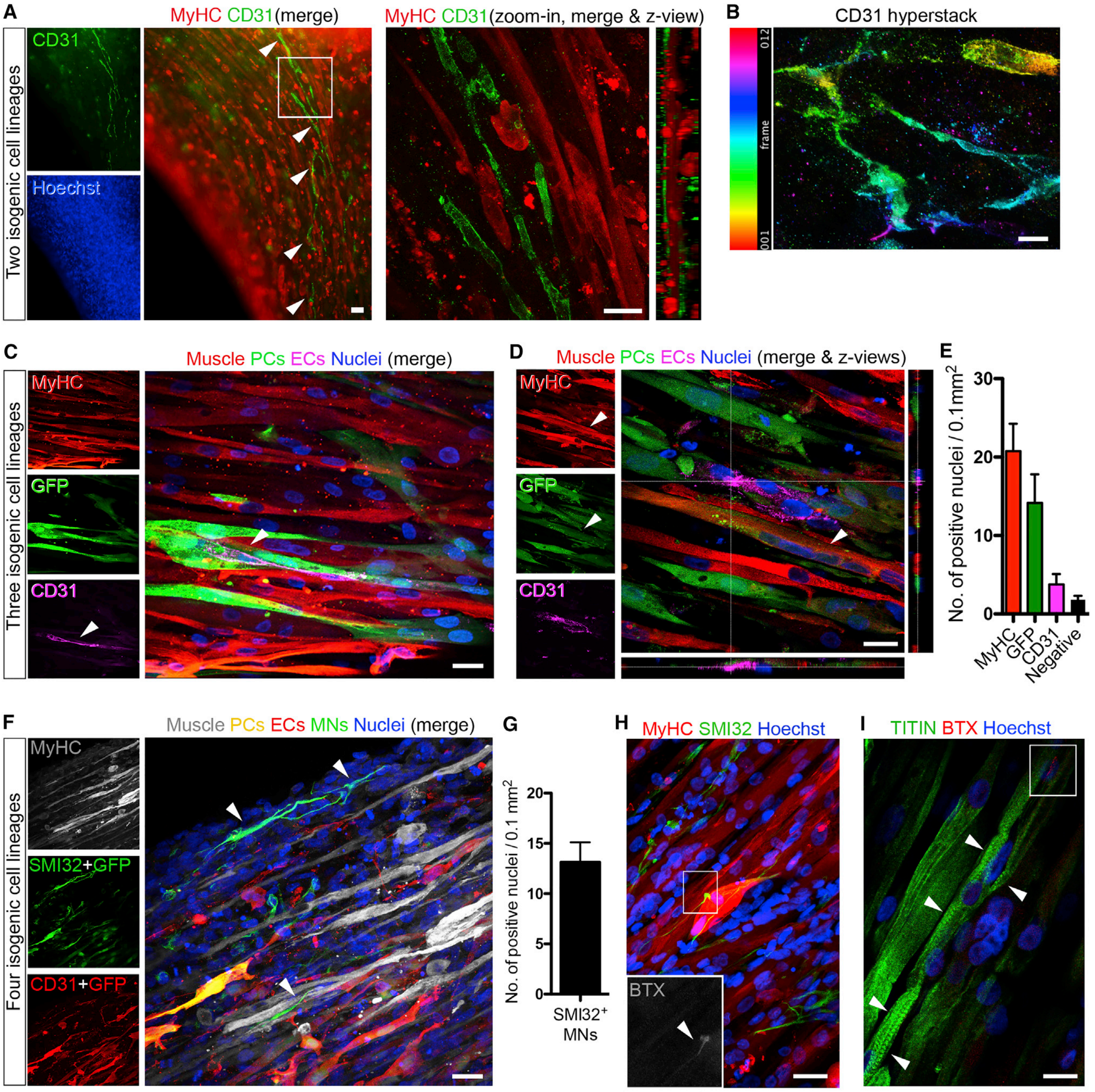

Figure 4. Multilineage Artificial Muscles Containing Isogenic hiPSC-Derived Vascular Cells and Motor Neurons

(A) Left panel: whole-mount immunofluorescence of an artificial muscle containing a self-organized isogenic network of hiPSC-derived CD31$^+$ endothelial cells (arrowheads). Right panel: higher-magnification confocal image of the boxed area showing lateral z views.

(B) Hyper-stack image (12 frames) processed with color-coding on CD31 staining (ImageJ) highlighting the 3D structure of the endothelial network. Frame thickness: 2 μm.

(C) Confocal images of whole-mount immunolabeling of a multilineage construct containing GFP$^+$ pericytes (PCs), displaying coexistence of myofibers (MyHC), ECs (CD31), and PCs. Arrowheads: CD31$^+$ ECs juxtaposed to PCs.

(D) Confocal image showing an additional example of a multilineage construct as in (C) with lateral z views. Arrowhead indicates a MyHC$^+$ and GFP$^+$ multinucleated myotube (see Discussion).

(E) Quantification of confocal images of tri-lineage artificial muscles showing the average number of MyHC$^+$ (muscle), GFP$^+$ (PCs), and CD31$^+$ (ECs) nuclei per 0.1 mm^2 field. Error bars: SEM. n = 10 images.

(F) Confocal immunofluorescence panel of multilineage 3D artificial muscle derived from WT hiPSCs containing isogenic myofibers, vascular cells (ECs and GFP$^+$ PCs), and motor neurons (SMI32). Color-coded combination enabled discrimination of the four cell types based upon the color of the merge. Arrowheads highlight two motor neurons showing multiple axon-like processes.

(legend continued on next page)

cells within hydrogels under tension and tested media that supported growth and differentiation of those lineages. After 10 days in culture, long (up to 0.9 mm) CD31$^+$ vessel-like formations coexisted in the same 3D environment close to isogenic myofibers (Figure 4A). The 3D nature of EC networks was visualized through hyper-stack images processed with depth color-coding for CD31 immunolabeling (Figure 4B). Due to the absence of unequivocal PC-specific markers (Armulik et al., 2011), hiPSC-derived PCs were transduced with a lentivirus encoding for GFP to allow their detection within artificial muscles. Immunofluorescence demonstrated alignment and coexistence of myofibers, ECs, and PCs within the same 3D environment (Figures 4C–4E). *In vivo* studies of muscle function in immunodeficient mice suggested enhanced force recovery only in muscles receiving multicellular versus single-lineage (i.e., only myofibers) artificial muscle implants (Figure S3B).

Another key cell type for skeletal muscle is the spinal motor neuron. Deriving primary human motor neurons is challenging; however, several protocols are available to differentiate them from hPSCs (Patani, 2016), including their coculture with primary myotubes (Steinbeck et al., 2016). Therefore, developing an isogenic human muscle-motor neurons platform would model neuromuscular disorders in a personalized fashion. To this aim, we differentiated hiPSCs into neural progenitors (Stacpoole et al., 2011) and further adapted our tri-lineage culture system to enable differentiation of neural precursors into motor neurons. We obtained stable 3D artificial muscle constructs containing four distinct isogenic cell types, i.e., myofibers, ECs, PCs, and SMI32+ cells with long axon-like processes resembling motor neurons spreading from hiPSC-derived neurospheres placed above the hydrogels (Figures 4F and 4G).

We next investigated generation of neuromuscular junctions through muscle-motor neuron bi-lineage models. Interactions between the two cell types were optimized by employing a differentiation paradigm that generates a highly enriched motor neuron population (Figure S3C) (Hall et al., 2017) and allows seeding of single-cell neural precursors within the hydrogel. DMD muscle constructs were made containing WT motor neurons, which showed alpha-bungarotoxin$^+$ acetylcholine receptors and striated myofibers with the sarcomeric protein Titin (Figures 4H and 4I), indicating a positive effect on myofibers' maturation exerted by motor neurons. Thus, we generated complex, 3D, multilineage, artificial skeletal muscle models from hiPSCs.

DISCUSSION

Use of hPSCs for tissue engineering and complex disease modeling is expanding, and exciting results have been obtained with hiPSC-derived organoids (Passier et al., 2016). Here we show that 3D constructs resembling skeletal muscle tissue can be generated by differentiating healthy donor and disease-specific hPSCs within fibrin hydrogels under unidirectional tension. We have generated artificial muscles from patients affected by severe forms of muscle diseases with different genetic inheritance, namely, Duchenne (X-linked), LGMD2D (autosomal recessive), and *LMNA*-related (autosomal dominant) muscular dystrophies. Our hiPSC-derived 3D platform recapitulated nuclear abnormalities characteristic of *LMNA*-related muscular dystrophies. Nuclear elongation was the most prominent abnormality, consistent with reports using *LMNA* mutant mice and primary human myoblasts (Bertrand et al., 2014; Nikolova et al., 2004), supporting the high fidelity of our 3D platform for modeling skeletal muscle laminopathies. We also provide proof of principle of hPSC-derived artificial muscle engraftment, laying the foundation for *in vivo* modeling and drug testing in humanized dystrophic muscles.

A major advantage of using hPSCs for muscle bioengineering is the ability to derive different cell types from the same cellular source, and here we provide evidence of generation of isogenic, multilineage, hPSC-derived artificial skeletal muscles. Although our data and published work (Rao et al., 2018) indicate that skeletal muscle properties can be observed in bundles of hPSC-derived myofibers, our results further indicate that maturation of fully functional artificial muscles might require contribution from other cellular lineages, such as vascular cells and motor neurons (Christov et al., 2007; Ecob-Prince et al., 1986; Kostallari et al., 2015; Martin et al., 2015). This could be particularly relevant for *in vitro* studies of non-muscle-specific defects in muscle disorders (Dabiré et al., 2012; Palladino et al., 2013) or to predict off-target effects of skeletal muscle-directed therapeutics. Artificial muscles containing vascular cells will likely improve engraftment upon implantation *in vivo*, via rapid anastomosis with the host circulation, in line with improved engraftment of vascularized muscle constructs derived from primary myoblasts (Perry et al., 2017; Quarta et al., 2017). Moreover, multilineage artificial muscles could also provide insights into human muscle regeneration dynamics, because we observed generation of GFP$^+$ myofibers in constructs in which only PCs had been transduced with a GFP-encoding lentiviral vector (Figure 4D). This suggests myogenic potential of PCs or their recruitment from differentiating muscle, similar to that described in mice with lineage tracing experiments (Dellavalle et al., 2011).

This platform can be further engineered to include other pluripotent derivatives, such as different muscle interstitial cells (Tedesco et al., 2017). Nonetheless, bioengineering an all-hPSC-derived muscle will require highly specialized culture conditions, potentially with a combination of transgene-based (e.g., Darabi et al., 2012; Tedesco et al., 2012) and transgene-free (e.g., Caron et al., 2016; Chal et al., 2015) differentiation methods, because culture conditions to maintain non-myogenic cells might interfere with transgene-free, small molecule-based myogenic differentiation of hPSCs. Further optimization of artificial muscles includes

(G) Quantification of the motor neurons (MNs) in quadruple lineage cultures shown in (F). Error bars: SEM. n = 7 images.

(H) Confocal immunofluorescence of a DMD artificial muscle construct containing non-isogenic WT SMI32+ motor neurons and showing alpha-bungarotoxin (BTX)$^+$ acetylcholine receptors (zoom in).

(I) Confocal immunofluorescence of a sister construct of (H) with aligned, multinucleated myofibers with sarcomeric TITIN+ striations (arrowheads) and BTX$^+$ acetylcholine receptors (red signal in white box).

Scale bars: (A and B) 25 μm; (C–F, H, and I) 10 μm. For additional information, see Figure S3.

improvement of maturation via chemical, electrical or optical stimulation and scaling down for high-throughput screening. Moreover, culture in autologous fibrinogen (de la Puente and Ludeña, 2014) would enable a highly personalized platform. Finally, human muscle models would reduce laboratory animal use for toxicity testing. In conclusion, this hPSC-derived artificial skeletal muscle platform could bring together regenerative medicine and drug development under the same translational technology, advancing knowledge on the pathogenesis and development of therapies for muscle diseases.

EXPERIMENTAL PROCEDURES

Generation of 3D Skeletal Muscle Constructs

15 hPSC lines (1 hESC line + 14 hiPSC lines) and 1 primary human myoblast line were used. Hydrogels were produced as published (Hansen et al., 2010) and according to the manufacturer's instructions (EHT Technologies, Hamburg). 10^6 myogenic cells pre-treated with ROCK inhibitor (10 μM; 1–2 hr) were used per construct (total volume: 120 μL). Artificial muscles were cultured at 37°C with 5% CO_2 supplementing the medium with 33 μg/μL aprotinin (Sigma, A3428) to prevent fibrinogen degradation. To induce myogenic differentiation, 1 μM 4-OH tamoxifen was added 48 hr after polymerization in proliferation medium and then 24 hr later in differentiation medium. For transgene-free myogenic differentiation (Genea Biocells), progenitors were cultured for 7 days in commitment medium before cells were combined with fibrin, switching to differentiation medium 2 days later. To generate $PAX7^+$ cells, myogenic commitment was induced for 2 days in standard monolayer culture conditions and then continued in 3D for 5 days before switching to differentiation medium; hydrogels were cultured for a total of 14 days. $Pax7^+$ cells were observed in 1 of 3 lines (NCRM1 iPSCs).

Triple-lineage constructs were made with the same method using a mix of 70% myogenic cells (7×10^5) and 30% vascular cells (6×10^4 ECs and 2.4×10^5 PCs). Muscles were cultured in a 1:1 mix of human iPSC-derived mesoangioblast-like inducible myogenic cells (HIDEM) proliferation medium (Maffioletti et al., 2015) and endothelial medium A (EC-SFM [endothelial cell-serum-free basal medium]; 1% platelet-poor, plasma-derived serum; 30 ng/mL vascular endothelial growth factor [VEGF]; 20 ng/mL basic fibroblast growth factor [bFGF]) for 48 hr and then in a 1:1 mix of HIDEM differentiation medium (Maffioletti et al., 2015) and endothelial medium B (Lonza, CC-3162) after the second tamoxifen administration. Muscles were cultured for 10 days at 37°C with 5% CO_2, changing the medium every other day. Human fibrinogen was kindly provided by Prof. H. Redl (LBG, Vienna).

Constructs containing four lineages (muscle + ECs + PCs + motor neurons) were made as described earlier; after 1 hr of polymerization, 6 neurospheres (neural progenitor cells [NPCs]) were decanted on top of the hydrogels using 10 μL of fibrin. After the second hour of polymerization, hydrogels were placed in media containing an equal ratio of Iscove's Modified Dulbecco's Medium (IMDM)-based HIDEM proliferation medium, EC-SFM (Orlova et al., 2014), and a chemically defined medium (Stacpoole et al., 2011), supplemented with heparin (5 μg/mL) and retinoic acid (0.1 μM). NPC differentiation to motor neurons was achieved through supplementation of the media with retinoic acid for 7 days and then with retinoic acid and purmorphamine (1 μM) until fixation at day 14.

To produce hydrogels containing myofibers (70%) and single-cell motor neurons (30%) (derived as per Hall et al., 2017), constructs were cultured for 48 hr using a 1:1 mix of HIDEM proliferation medium with 1% fetal bovine serum (FBS) and neural precursor medium (Shi et al., 2012). The same mix was supplemented with 4-OH tamoxifen and 0.1 μM γ-secretase inhibitor (Sigma, L1790) to induce myogenic and neural differentiation. After 24 hr, medium was changed to administer the second tamoxifen pulse. Hydrogels were kept in culture for 15 days at 37°C with 5% CO_2, changing the medium mix with 0.1 μM γ-secretase inhibitor every day. At day 5, the medium was supplemented with agrin (R&D Systems, 550-AG/CF) to promote neuromuscular junction formation. Agrin concentrations of 0.1, 0.5, and 1 nM were used over the 3 initial days, and the final concentration was kept until fixation of constructs. Additional information can be found in Supplemental Experimental Procedures.

Analysis of Nuclear Abnormalities in *LMNA* Mutant Artificial Muscles

LMNA mutant iPSCs derived from three patients affected by skeletal muscle laminopathies (LGMD1B and L-CMD) were provided by Cellular Dynamics International (CDI; http://www.cellulardynamics.com) and Cure Congenital Muscular Dystrophy (CureCMD; http://www.curecmd.org). iPSCs were derived by CDI using episomal vectors from samples provided by CureCMD, which holds patients' clinical information. Pluripotency was tested by CDI with a proprietary set of genes. *LMNA* mutant iPSCs had three heterozygous dominant mutations: p.K32del and p.L35P located in *LMNA* exon 1 and p.R249W in *LMNA* exon 4.

Hydrogels were fixed with 4% paraformaldehyde (PFA) for 3 hr at 4°C followed by 6 hr of blocking at 4°C (10% FBS, 1% BSA, and 0.5% Triton X-100 in 0.05 M Tris-buffered saline [TBS]) before immunolabeling with rabbit anti-Desmin (1:150) (Sigma, D8281), mouse anti-LAMIN A/C (1:100) (Novocastra NCL-LAM), and goat anti-Emerin (1:50) (Santa Cruz, sc8086) antibodies overnight at 4°C in TBS, 1% BSA, and 0.5% Triton X-100. The next day, hydrogels were washed with TBS 6 times hourly and incubated overnight with Hoechst 33342 (Sigma, B2261) plus species-specific secondary antibodies (Alexa Fluor 488, 546, and 647) (Thermo Fisher Scientific). The following day, hydrogels were washed 6 times with TBS and embedded in mounting medium (Dako, S3023A) on glass slides. A confocal microscope (Leica, SPE2) was used for imaging, using 95× magnification to take 5 to 9 z stacks (step size: 0.5 μm) of randomly selected regions (final thickness: 12–82 μm). The z stacks were 3D reconstructed and analyzed based on LAMIN A/C immunolabeling using Imaris 8.4.1 software (Bitplane). Nuclei were analyzed by scoring the number of abnormalities per field and by measuring major axis length. Oval or slightly elongated nuclei were scored as normal. Three *LMNA* mutant patients and three controls were quantified. A minimum of 45 nuclei per hydrogel per experiment across 8 random fields was scored. Reproducibility was validated by 3 independent operators (one researcher was blinded) (Table S1). Normal distribution of nuclear abnormalities was tested using the D'Agostino and Pearson test and statistical testing compared the *LMNA* mutant group (N = 6; 3 cell populations in 2 experiments) versus the non-mutant group (N = 6; 3 cell populations, 2 experiments) using Mann-Whitney U test, because one cell population per group did not have a normal distribution of values.

Ethics

Work with human cells was performed under approval of the National Health Service (NHS) Health Research Authority Research Ethics Committee (reference No. 13/LO/1826) and Integrated Research Application System (IRAS) project (ID No. 141100) and, for motor neuron work, according to approved regulations and guidelines by University College London Hospital's National Hospital for Neurology and Neurosurgery and University College London's (UCL) Institute of Neurology joint research ethics committee (09/0272). Use of hESCs was approved by the Steering Committee for the UK Stem Cell Bank and for the use of stem cell lines (SCSC12-46 and SCSC13-14). Procedures involving animals were approved by the UK Home Office according to Animals (Scientific Procedures) Act (ASPA) regulations and performed under PPL 70/8566.

Statistics

Values are mean ± SD or SEM (as specified). Specific N or n values and statistical tests are indicated in figure legends. Data were analyzed with Microsoft Excel and GraphPad Prism.

DATA AND SOFTWARE AVAILABILITY

The raw data reported in this paper have been deposited in Mendeley Data and are available at https://doi.org/10.17632/d826fxhr3b.1.

SUPPLEMENTAL INFORMATION

Supplemental Information includes Supplemental Experimental Procedures, three figures, one table, and two videos and can be found with this article online at https://doi.org/10.1016/j.celrep.2018.03.091.

ACKNOWLEDGMENTS

We thank G. Cossu, H. Redl, D. Seliktar, and the Biodesign Consortium (EU FP7 project 262948) for initial support and materials. We are grateful to CureCMD, Cellular Dynamics International, Inc., P. Andrews, M. Oshimura, and T. VandenDriessche for providing hPSCs; to J. Morgan for titin antibody; and to S. Benedetti, M. Plotczyk, L. Miller, and lab members for feedback and assistance. The Graphical Abstract was created using Servier Medical Art (https://smart.servier.com) in accordance with a Creative Commons Attribution 3.0 Unported License (https://creativecommons.org/licenses/by/3.0/). This work received funding from the European Union's 7th Framework Program for research, technological development, and demonstration under grant agreement 602423 (PluriMes) and from the BBSRC LIDo program (BB/J014567/1 and BB/M009513/1 to H.S.-S., L.P., P.S.Z., and F.S.T.). F.S.T. is funded by a National Institute for Health Research (NIHR) Academic Clinical Fellowship in Pediatrics (ACF-2015-18-001), and he is grateful to F. Muntoni for mentorship and expert advice. This paper presents independent research funded by the NIHR. The views expressed are those of the authors and not necessarily those of the National Health Service (NHS), the NIHR, or the Department of Health. Work in the Tedesco lab has also received support from the Innovative Medicines Initiative Joint Undertaking under grant agreement 115582 (EBiSC; European Union's 7th Framework Program and EFPIA companies), the MRC (MR/R014108/1, MR/L002752/1, and MR/J006785/1), Takeda New Frontier Science, Fundació La Marató de TV3 (201440.30.31.32), and Muscular Dystrophy UK (RA4/3023/1 and 17GRO-PS48-0093-1).

AUTHOR CONTRIBUTIONS

Conceptualization, F.S.T. and S.M.M.; Methodology & Investigation, S.M.M., S.S., A.B.H.H., I.M., L.P., L.A.M., H.S.-S., O.C., K.E.W., G.F., J.S.M., G.E.T., V.N.K., M.R., W.W., and F.S.T.; Data Analysis & Discussion, S.M.M., S.S., A.B.H.H., I.M., L.P., L.A.M., H.S.-S., O.C., K.E.W., V.N.K., M.K., M.R.D., R.P., P.S.Z., D.J.W., T.E., and F.S.T.; Writing – Original Draft, Review, & Editing, S.M.M. and F.S.T., with inputs from all coauthors; Final Manuscript, Main Funding, & Coordination, F.S.T.

DECLARATION OF INTERESTS

F.S.T. was principal investigator on a research grant from Takeda New Frontier Science Program (2014–2016), received speaking and consulting fees from Takeda and Sanofi-Genzyme (via UCL Consultants), and has a collaboration with GSK (unrelated research project, via BBSRC ICP BB/N503915/1). I.M. and T.E. are cofounders of EHT Technologies, GmbH. All other authors declare no competing interests.

Received: November 15, 2016
Revised: February 21, 2018
Accepted: March 20, 2018
Published: April 17, 2018

REFERENCES

Armulik, A., Genové, G., and Betsholtz, C. (2011). Pericytes: developmental, physiological, and pathological perspectives, problems, and promises. Dev. Cell *21*, 193–215.

Bertrand, A.T., Ziaei, S., Ehret, C., Duchemin, H., Mamchaoui, K., Bigot, A., Mayer, M., Quijano-Roy, S., Desguerre, I., Lainé, J., et al. (2014). Cellular microenvironments reveal defective mechanosensing responses and elevated YAP signaling in LMNA-mutated muscle precursors. J. Cell Sci. *127*, 2873–2884.

Caron, L., Kher, D., Lee, K.L., McKernan, R., Dumevska, B., Hidalgo, A., Li, J., Yang, H., Main, H., Ferri, G., et al. (2016). A Human Pluripotent Stem Cell Model of Facioscapulohumeral Muscular Dystrophy-Affected Skeletal Muscles. Stem Cells Transl. Med. *5*, 1145–1161.

Carosio, S., Barberi, L., Rizzuto, E., Nicoletti, C., Del Prete, Z., and Musarò, A. (2013). Generation of eX vivo-vascularized Muscle Engineered Tissue (X-MET). Sci. Rep. *3*, 1420.

Chal, J., Oginuma, M., Al Tanoury, Z., Gobert, B., Sumara, O., Hick, A., Bousson, F., Zidouni, Y., Mursch, C., Moncuquet, P., et al. (2015). Differentiation of pluripotent stem cells to muscle fiber to model Duchenne muscular dystrophy. Nat. Biotechnol. *33*, 962–969.

Chiron, S., Tomczak, C., Duperray, A., Lainé, J., Bonne, G., Eder, A., Hansen, A., Eschenhagen, T., Verdier, C., and Coirault, C. (2012). Complex interactions between human myoblasts and the surrounding 3D fibrin-based matrix. PLoS ONE *7*, e36173.

Christov, C., Chrétien, F., Abou-Khalil, R., Bassez, G., Vallet, G., Authier, F.J., Bassaglia, Y., Shinin, V., Tajbakhsh, S., Chazaud, B., and Gherardi, R.K. (2007). Muscle satellite cells and endothelial cells: close neighbors and privileged partners. Mol. Biol. Cell *18*, 1397–1409.

Corona, B.T., Ward, C.L., Baker, H.B., Walters, T.J., and Christ, G.J. (2014). Implantation of *in vitro* tissue engineered muscle repair constructs and bladder acellular matrices partially restore *in vivo* skeletal muscle function in a rat model of volumetric muscle loss injury. Tissue Eng. Part A *20*, 705–715.

Criswell, T.L., Corona, B.T., Wang, Z., Zhou, Y., Niu, G., Xu, Y., Christ, G.J., and Soker, S. (2013). The role of endothelial cells in myofiber differentiation and the vascularization and innervation of bioengineered muscle tissue *in vivo*. Biomaterials *34*, 140–149.

Dabiré, H., Barthélémy, I., Blanchard-Gutton, N., Sambin, L., Sampedrano, C.C., Gouni, V., Unterfinger, Y., Aguilar, P., Thibaud, J.L., Ghaleh, B., et al. (2012). Vascular endothelial dysfunction in Duchenne muscular dystrophy is restored by bradykinin through upregulation of eNOS and nNOS. Basic Res. Cardiol. *107*, 240.

Darabi, R., Arpke, R.W., Irion, S., Dimos, J.T., Grskovic, M., Kyba, M., and Perlingeiro, R.C. (2012). Human ES- and iPS-derived myogenic progenitors restore DYSTROPHIN and improve contractility upon transplantation in dystrophic mice. Cell Stem Cell *10*, 610–619.

de la Puente, P., and Ludeña, D. (2014). Cell culture in autologous fibrin scaffolds for applications in tissue engineering. Exp. Cell Res. *322*, 1–11.

Dellavalle, A., Maroli, G., Covarello, D., Azzoni, E., Innocenzi, A., Perani, L., Antonini, S., Sambasivan, R., Brunelli, S., Tajbakhsh, S., and Cossu, G. (2011). Pericytes resident in postnatal skeletal muscle differentiate into muscle fibres and generate satellite cells. Nat. Commun. *2*, 499.

Ecob-Prince, M.S., Jenkison, M., Butler-Browne, G.S., and Whalen, R.G. (1986). Neonatal and adult myosin heavy chain isoforms in a nerve-muscle culture system. J. Cell Biol. *103*, 995–1005.

Fatehullah, A., Tan, S.H., and Barker, N. (2016). Organoids as an *in vitro* model of human development and disease. Nat. Cell Biol. *18*, 246–254.

Fuoco, C., Rizzi, R., Biondo, A., Longa, E., Mascaro, A., Shapira-Schweitzer, K., Kossovar, O., Benedetti, S., Salvatori, M.L., Santoleri, S., et al. (2015). *In vivo* generation of a mature and functional artificial skeletal muscle. EMBO Mol. Med. *7*, 411–422.

Gholobova, D., Decroix, L., Van Muylder, V., Desender, L., Gerard, M., Carpentier, G., Vandenburgh, H., and Thorrez, L. (2015). Endothelial Network Formation Within Human Tissue-Engineered Skeletal Muscle. Tissue Eng. Part A *21*, 2548–2558.

Giacomelli, E., Bellin, M., Sala, L., van Meer, B.J., Tertoolen, L.G., Orlova, V.V., and Mummery, C.L. (2017). Three-dimensional cardiac microtissues composed of cardiomyocytes and endothelial cells co-differentiated from human pluripotent stem cells. Development *144*, 1008–1017.

Grogan, B.F., and Hsu, J.R.; Skeletal Trauma Research Consortium (2011). Volumetric muscle loss. J. Am. Acad. Orthop. Surg. *19* (*Suppl 1*), S35–S37.

Hall, C.E., Yao, Z., Choi, M., Tyzack, G.E., Serio, A., Luisier, R., Harley, J., Preza, E., Arber, C., Crisp, S.J., et al. (2017). Progressive Motor Neuron Pathology and the Role of Astrocytes in a Human Stem Cell Model of VCP-Related ALS. Cell Rep. *19*, 1739–1749.

Hansen, A., Eder, A., Bönstrup, M., Flato, M., Mewe, M., Schaaf, S., Aksehirlioglu, B., Schwoerer, A.P., Uebeler, J., and Eschenhagen, T. (2010).

Development of a drug screening platform based on engineered heart tissue. Circ. Res. *107*, 35–44.

Huang, Y.C., Dennis, R.G., Larkin, L., and Baar, K. (2005). Rapid formation of functional muscle *in vitro* using fibrin gels. J. Appl. Physiol. *98*, 706–713.

Inoue, H., Nagata, N., Kurokawa, H., and Yamanaka, S. (2014). iPS cells: a game changer for future medicine. EMBO J. *33*, 409–417.

Juhas, M., Engelmayr, G.C., Jr., Fontanella, A.N., Palmer, G.M., and Bursac, N. (2014). Biomimetic engineered muscle with capacity for vascular integration and functional maturation *in vivo*. Proc. Natl. Acad. Sci. USA *111*, 5508–5513.

Kirschner, J., and Lochmüller, H. (2011). Sarcoglycanopathies. Handb. Clin. Neurol. *101*, 41–46.

Koffler, J., Kaufman-Francis, K., Shandalov, Y., Egozi, D., Pavlov, D.A., Landesberg, A., and Levenberg, S. (2011). Improved vascular organization enhances functional integration of engineered skeletal muscle grafts. Proc. Natl. Acad. Sci. USA *108*, 14789–14794.

Kostallari, E., Baba-Amer, Y., Alonso-Martin, S., Ngoh, P., Relaix, F., Lafuste, P., and Gherardi, R.K. (2015). Pericytes in the myovascular niche promote post-natal myofiber growth and satellite cell quiescence. Development *142*, 1242–1253.

Lancaster, M.A., and Knoblich, J.A. (2014). Organogenesis in a dish: modeling development and disease using organoid technologies. Science *345*, 1247125.

Levenberg, S., Rouwkema, J., Macdonald, M., Garfein, E.S., Kohane, D.S., Darland, D.C., Marini, R., van Blitterswijk, C.A., Mulligan, R.C., D'Amore, P.A., and Langer, R. (2005). Engineering vascularized skeletal muscle tissue. Nat. Biotechnol. *23*, 879–884.

Machingal, M.A., Corona, B.T., Walters, T.J., Kesireddy, V., Koval, C.N., Dannahower, A., Zhao, W., Yoo, J.J., and Christ, G.J. (2011). A tissue-engineered muscle repair construct for functional restoration of an irrecoverable muscle injury in a murine model. Tissue Eng. Part A *17*, 2291–2303.

Madden, L., Juhas, M., Kraus, W.E., Truskey, G.A., and Bursac, N. (2015). Bioengineered human myobundles mimic clinical responses of skeletal muscle to drugs. eLife *4*, e04885.

Maffioletti, S.M., Gerli, M.F., Ragazzi, M., Dastidar, S., Benedetti, S., Loperfido, M., VandenDriessche, T., Chuah, M.K., and Tedesco, F.S. (2015). Efficient derivation and inducible differentiation of expandable skeletal myogenic cells from human ES and patient-specific iPS cells. Nat. Protoc. *10*, 941–958.

Maggi, L., Carboni, N., and Bernasconi, P. (2016). Skeletal Muscle Laminopathies: A Review of Clinical and Molecular Features. Cells *5*, E33.

Martin, N.R., Passey, S.L., Player, D.J., Mudera, V., Baar, K., Greensmith, L., and Lewis, M.P. (2015). Neuromuscular Junction Formation in Tissue-Engineered Skeletal Muscle Augments Contractile Function and Improves Cytoskeletal Organization. Tissue Eng. Part A *21*, 2595–2604.

Mercuri, E., and Muntoni, F. (2013). Muscular dystrophies. Lancet *381*, 845–860.

Nikolova, V., Leimena, C., McMahon, A.C., Tan, J.C., Chandar, S., Jogia, D., Kesteven, S.H., Michalicek, J., Otway, R., Verheyen, F., et al. (2004). Defects in nuclear structure and function promote dilated cardiomyopathy in lamin A/C-deficient mice. J. Clin. Invest. *113*, 357–369.

Orlova, V.V., van den Hil, F.E., Petrus-Reurer, S., Drabsch, Y., Ten Dijke, P., and Mummery, C.L. (2014). Generation, expansion and functional analysis of endothelial cells and pericytes derived from human pluripotent stem cells. Nat. Protoc. *9*, 1514–1531.

Palladino, M., Gatto, I., Neri, V., Straino, S., Smith, R.C., Silver, M., Gaetani, E., Marcantoni, M., Giarretta, I., Stigliano, E., et al. (2013). Angiogenic impairment of the vascular endothelium: a novel mechanism and potential therapeutic target in muscular dystrophy. Arterioscler. Thromb. Vasc. Biol. *33*, 2867–2876.

Park, Y.E., Hayashi, Y.K., Goto, K., Komaki, H., Hayashi, Y., Inuzuka, T., Noguchi, S., Nonaka, I., and Nishino, I. (2009). Nuclear changes in skeletal muscle extend to satellite cells in autosomal dominant Emery-Dreifuss muscular dystrophy/limb-girdle muscular dystrophy 1B. Neuromuscul. Disord. *19*, 29–36.

Passier, R., Orlova, V., and Mummery, C. (2016). Complex Tissue and Disease Modeling using hiPSCs. Cell Stem Cell *18*, 309–321.

Patani, R. (2016). Generating Diverse Spinal Motor Neuron Subtypes from Human Pluripotent Stem Cells. Stem Cells Int. *2016*, 1036974.

Perry, L., Flugelman, M.Y., and Levenberg, S. (2017). Elderly Patient-Derived Endothelial Cells for Vascularization of Engineered Muscle. Mol. Ther. *25*, 935–948.

Powell, C., Shansky, J., Del Tatto, M., Forman, D.E., Hennessey, J., Sullivan, K., Zielinski, B.A., and Vandenburgh, H.H. (1999). Tissue-engineered human bioartificial muscles expressing a foreign recombinant protein for gene therapy. Hum. Gene Ther. *10*, 565–577.

Quarta, M., Cromie, M., Chacon, R., Blonigan, J., Garcia, V., Akimenko, I., Hamer, M., Paine, P., Stok, M., Shrager, J.B., and Rando, T.A. (2017). Bioengineered constructs combined with exercise enhance stem cell-mediated treatment of volumetric muscle loss. Nat. Commun. *8*, 15613.

Rao, L., Qian, Y., Khodabukus, A., Ribar, T., and Bursac, N. (2018). Engineering human pluripotent stem cells into a functional skeletal muscle tissue. Nat. Commun. *9*, 126.

Scharner, J., Figeac, N., Ellis, J.A., and Zammit, P.S. (2015). Ameliorating pathogenesis by removing an exon containing a missense mutation: a potential exon-skipping therapy for laminopathies. Gene Ther. *22*, 503–515.

Shandalov, Y., Egozi, D., Koffler, J., Dado-Rosenfeld, D., Ben-Shimol, D., Freiman, A., Shor, E., Kabala, A., and Levenberg, S. (2014). An engineered muscle flap for reconstruction of large soft tissue defects. Proc. Natl. Acad. Sci. USA *111*, 6010–6015.

Shi, Y., Kirwan, P., and Livesey, F.J. (2012). Directed differentiation of human pluripotent stem cells to cerebral cortex neurons and neural networks. Nat. Protoc. *7*, 1836–1846.

Stacpoole, S.R., Bilican, B., Webber, D.J., Luzhynskaya, A., He, X.L., Compston, A., Karadottir, R., Franklin, R.J., and Chandran, S. (2011). Efficient derivation of NPCs, spinal motor neurons and midbrain dopaminergic neurons from hESCs at 3% oxygen. Nat. Protoc. *6*, 1229–1240.

Steinbeck, J.A., Jaiswal, M.K., Calder, E.L., Kishinevsky, S., Weishaupt, A., Toyka, K.V., Goldstein, P.A., and Studer, L. (2016). Functional Connectivity under Optogenetic Control Allows Modeling of Human Neuromuscular Disease. Cell Stem Cell *18*, 134–143.

Tchao, J., Kim, J.J., Lin, B., Salama, G., Lo, C.W., Yang, L., and Tobita, K. (2013). Engineered Human Muscle Tissue from Skeletal Muscle Derived Stem Cells and Induced Pluripotent Stem Cell Derived Cardiac Cells. Int. J. Tissue Eng. *2013*, 198762.

Tedesco, F.S., Dellavalle, A., Diaz-Manera, J., Messina, G., and Cossu, G. (2010). Repairing skeletal muscle: regenerative potential of skeletal muscle stem cells. J. Clin. Invest. *120*, 11–19.

Tedesco, F.S., Gerli, M.F., Perani, L., Benedetti, S., Ungaro, F., Cassano, M., Antonini, S., Tagliafico, E., Artusi, V., Longa, E., et al. (2012). Transplantation of genetically corrected human iPSC-derived progenitors in mice with limb-girdle muscular dystrophy. Sci. Transl. Med. *4*, 140ra89.

Tedesco, F.S., Moyle, L.A., and Perdiguero, E. (2017). Muscle Interstitial Cells: A Brief Field Guide to Non-satellite Cell Populations in Skeletal Muscle. Methods Mol. Biol. *1556*, 129–147.

VanDusen, K.W., Syverud, B.C., Williams, M.L., Lee, J.D., and Larkin, L.M. (2014). Engineered skeletal muscle units for repair of volumetric muscle loss in the tibialis anterior muscle of a rat. Tissue Eng. Part A *20*, 2920–2930.

Worman, H.J. (2012). Nuclear lamins and laminopathies. J. Pathol. *226*, 316–325.

Cell Reports, Volume *23*

Supplemental Information

Three-Dimensional Human iPSC-Derived Artificial Skeletal Muscles Model Muscular Dystrophies and Enable Multilineage Tissue Engineering

Sara Martina Maffioletti, Shilpita Sarcar, Alexander B.H. Henderson, Ingra Mannhardt, Luca Pinton, Louise Anne Moyle, Heather Steele-Stallard, Ornella Cappellari, Kim E. Wells, Giulia Ferrari, Jamie S. Mitchell, Giulia E. Tyzack, Vassilios N. Kotiadis, Moustafa Khedr, Martina Ragazzi, Weixin Wang, Michael R. Duchen, Rickie Patani, Peter S. Zammit, Dominic J. Wells, Thomas Eschenhagen, and Francesco Saverio Tedesco

SUPPLEMENTAL FIGURES, TABLE AND VIDEOS

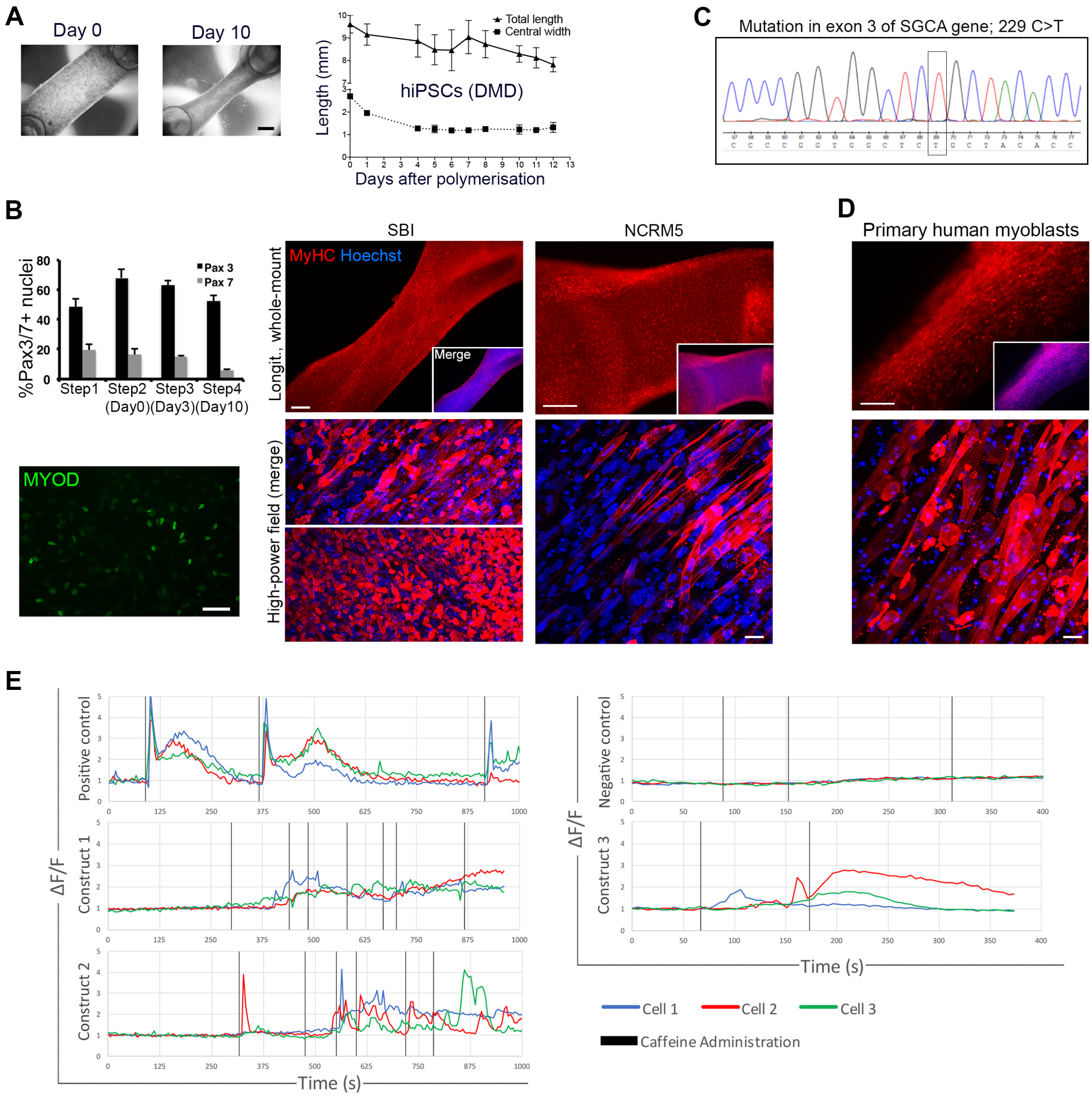

Figure S1. Additional characterization of hPSC-derived 3D artificial muscle constructs, related to Figure 2. (A) Representative phase contrast images of the morphology of cellularized hydrogels from a dystrophic patient (DMD) after polymerization (day 0) and after 10 days in culture. Gels undergo remodeling over time, as also shown by the graphs displaying shortening and thinning in culture. Data plotted as mean ± SD, N = 2 to 4 / point / line. **(B)** Generation of 3D muscle constructs from small-molecule based, transgene-free differentiation of hiPSCs as in (Caron et al., 2016). Histograms on the left show the percentage of PAX3^{+} and

$PAX7^+$ cells during monolayer differentiation (quantified by immunofluorescence staining). Bottom left immunofluorescence staining shows nuclear MYOD, demonstrating skeletal myogenic commitment the cultured cells. The two vertical immunofluorescence panels show MyHC staining as shown in Figure 1A in two additional WT iPSC lines. Some hiPSC lines (such as SBI) occasionally showed suboptimal alignment of myotubes in some gels, as shown in the bottom left high power field image. **(C)** Sequencing electropherogram of differentiated LGDM2D-iPSCs used for the experiments confirming the pathological point mutation in exon 3 of the alpha sarcoglycan (SGCA) gene. **(D)** Immunofluorescence panel as Figure 2A showing comparable differentiation and myofiber alignment in artificial muscle constructs generated using primary biopsy-derived human myoblasts. **(E)** Traces showing analysis of calcium transients in muscle constructs loaded with the fluorescent Ca^{2+} indicator Fluo-4AM upon caffeine administration. Three examples of representative responding cells per sample are shown. Vertical back lines indicate time of caffeine administration (10 mM). Three representative constructs out of the 4 tested are shown. Positive control: monolayer of human myotubes; negative control: undifferentiated cells (same line used in the constructs). ΔF/F: measured fluorescence relative to the average resting fluorescence of each point, before the administration of caffeine. Image analysis was conducted with ImageJ and Microsoft Excel was used to generate the graphs. Additional details in Supplemental Experimental Procedures. Scale bars: (A) 1mm (C) 100 μm MYOD and 400 μm others; (D) 400 μm.

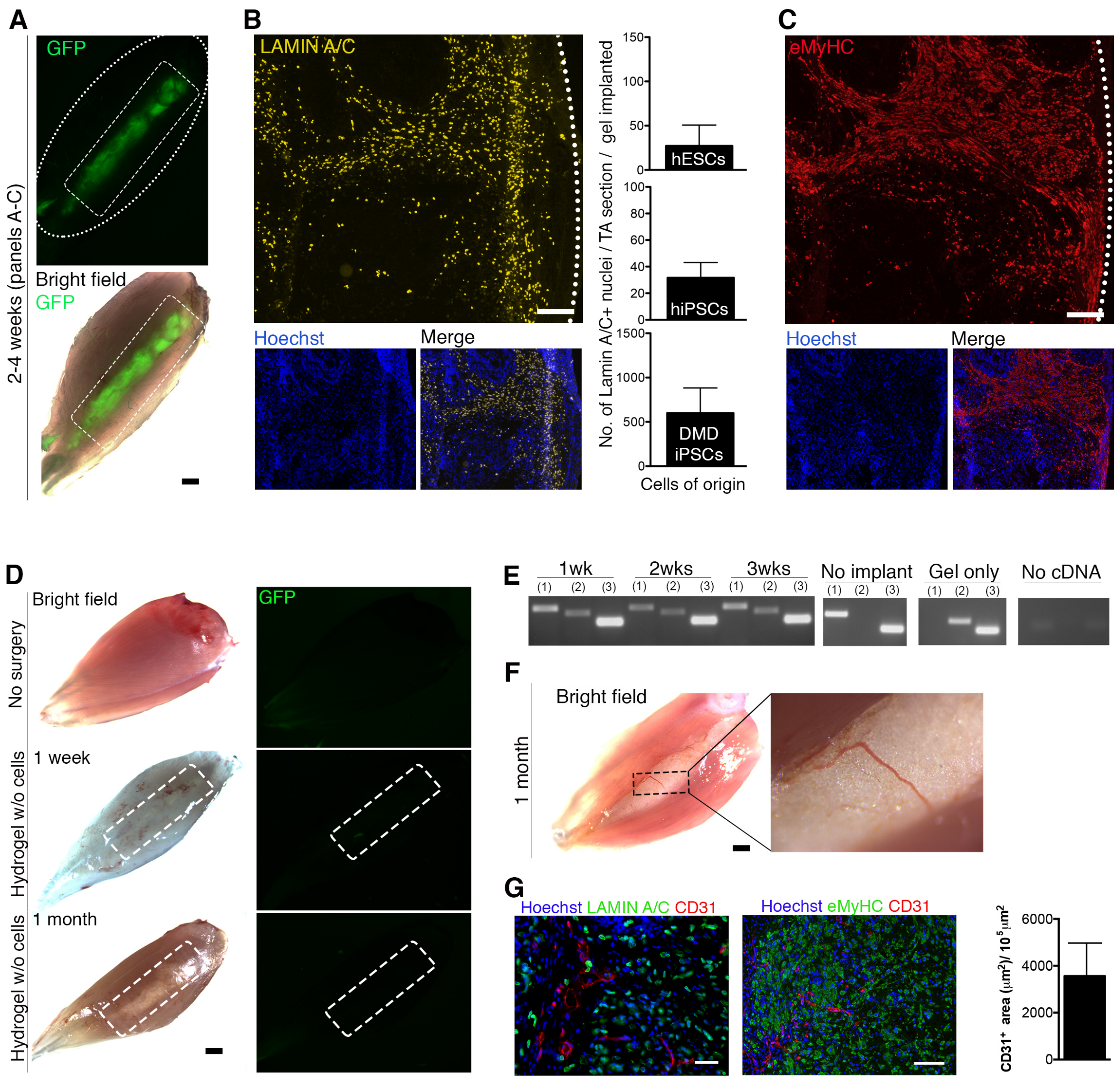

Figure S2. ***In vivo*** **implantation, engraftment and vascularization of hPSC-derived artificial muscles in immunodeficient mice, related to Figure 2.**

(A) Representative images of a whole tibialis anterior (TA) muscle 2-4 weeks after implantation in NSG mice. GFP$^+$ myogenic cells were used to generate artificial muscles. The white dashed squares and ovals indicate the artificial muscle and the mouse TA respectively. **(B)** Immunofluorescence images highlight the presence of engrafted human nuclei (LAMIN A/C$^+$) in transverse sections of TA muscles 2-4 weeks after implantation Data are from three different healthy or dystrophic cell lines; mean ± SD per TA section (normalized per implanted hydrogel): hESCs 27.14 ± 23.53; hiPSCs 31.59 ± 11.50; DMD hiPSCs 599.13 ± 284.06). Bar graphs on the right show quantification of the number of human nuclei in each muscle section. Bars

show mean ± SD; N = 7 for DMD, 3 for hiPSCs and 5 for hESCs. **(C)** Immunofluorescence staining for embryonic MyHC (eMyHC) of a serial section of the muscle shown in (B). **(D)** Images of whole TA muscles 1 week and 1 month after implantation of hydrogels without cells (middle and bottom images). A control muscle (no surgery) is shown at the top. The hydrogels do not display any autofluorescence in the GFP channel. **(E)** RT-PCR showing expression of alpha sarcoglycan (SGCA; 2) in the transplanted mouse TA at different time points. Control muscles only express mouse Sgca (1) whereas human artificial muscles cultured *in vitro* only express human SGCA. GAPDH was used as loading control (3). **(F)** Bright field images of a freshly explanted TA muscle 1 month after implantation showing blood vessels crossing the implant. **(G)** Immunofluorescence staining of human nuclei (lamin A/C), newly generated myofibres (eMyHC) and endothelial cells (CD31) within the transplanted artificial muscle in TA transverse sections 1-2 weeks after surgery. The area of $CD31^+$ structures present within the implant is quantified in the histogram on the right, showing the mean area normalized per 10^5 μm^2. 300 $CD31^+$ structures in total were measured in 5 pictures. Error bars: SD. Scale bars: (A,D,F) 1.5 mm; (B,C) 200 µm; (G) 50 µm left and 100 µm right.

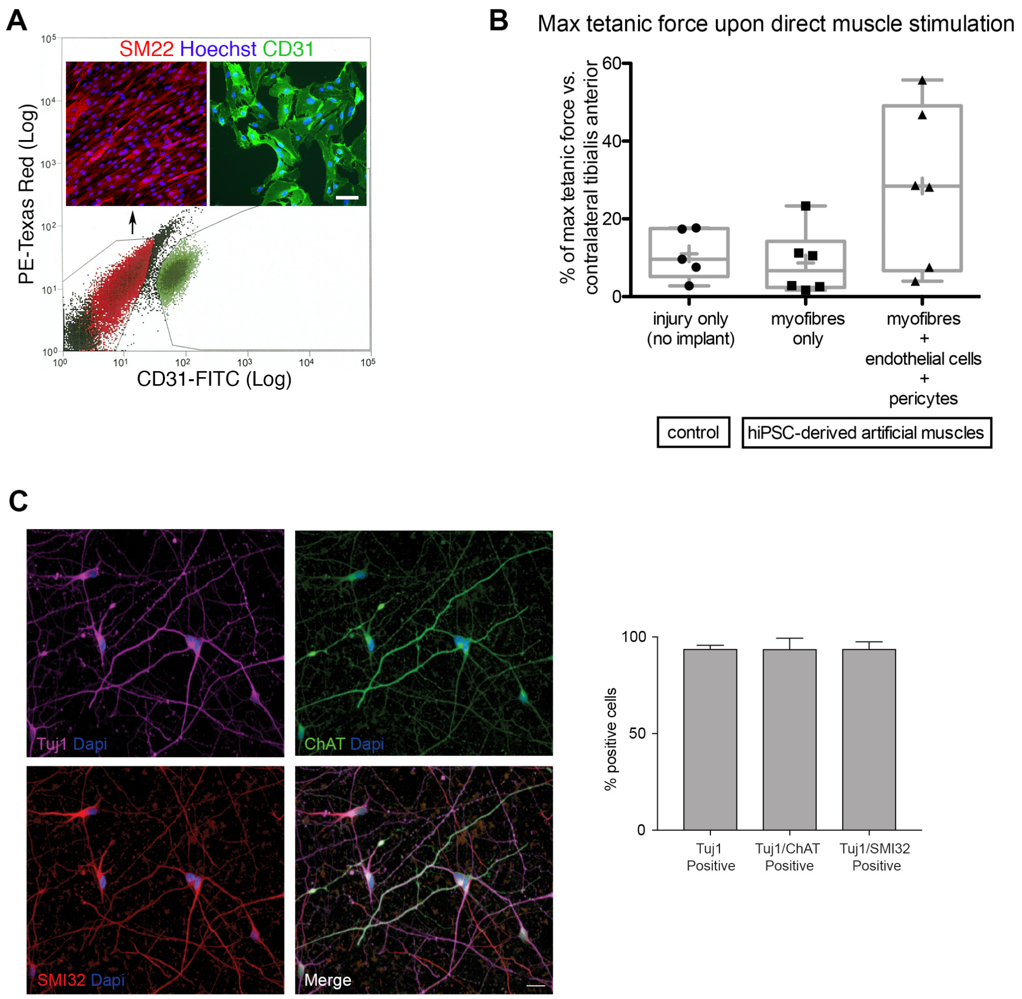

Figure S3. Multilineage hiPSC-derived skeletal muscle constructs: purification of vascular cells, assessment of muscle function *in vivo* and characterization of motor neurons, related to Figure 4. **(A)** FACS plot depicting isolation of $CD31^+$ endothelial cells (ECs) and $CD31^-$ pericytes (PCs; immunolabeled also with SM22) derived from isogenic iPSCs used for muscle generation. Details in Experimental Procedures. **(B)** Scatter plot graph (black symbols) with superimposed box-and-whiskers (gray) showing percentage of maximum tetanic force of tibialis anterior muscles (relative to uninjured contralateral muscles) of NSG mice implanted with hiPSC-derived artificial muscles (containing only myofibres or multicellular, i.e. myofibres + ECs + PCs; control: injury and no implant). One-way ANOVA, P = 0.0521; N = 17 mice (corresponding to each dot in the graph). Kruskal-Wallis test was also performed taking into account the N values per group and showed a P value of 0.127. Boxes: 25th to 75th percentiles; horizontal lines: median; "+" symbol: mean; whiskers: min to max values. **(C)** Efficiency of motor neuron (MN) generation

from hiPSCs assessed by immunofluorescence. Cultures were stained for the pan-neuronal marker Tuj1 (pink) and two MN-specific markers: choline acetyltransferase (ChAT) and SMI32. Data representative of differentiation from four different hiPSC lines, of which one was used in 3D co-culture experiments (ThermoFisher A18945). Nuclei were counterstained with Dapi (blue). Scale bars: 20 μm. Error bars: SD.

OPERATOR 1

	Elongated Nuclei	Nuclei with Blebs	Deformed Nuclei	Abnormal Nuclei	Normal Nuclei	Total Number of Nuclei
Control 1 (WT hiPSCs)	5.5% (8)	0% (0)	0.7% (1)	6.2% (9)	93.8% (137)	146
Control 2 (LGMD2D)	1.1% (1)	0% (0)	4.3% (4)	5.4% (5)	94.6% (88)	93
Control 3 (hESCs)	3.9% (4)	0% (0)	6.9% (7)	10.8% (11)	89.2% (91)	102
K32del	5.3% (5)	0% (0)	12.8% (12)	18.1% (17)	81.9% (77)	94
L35P	40.2% (39)	4.1% (4)	8.3% (8)	52.6% (51)	47.4% (46)	97
R249W	23.2% (36)	0% (0)	11% (17)	34.2% (53)	65.8% (102)	155

OPERATOR 2

	Elongated Nuclei	Nuclei with Blebs	Deformed Nuclei	Abnormal Nuclei	Normal Nuclei	Total Number of Nuclei
Control 1 (WT hiPSCs)	8.1 % (16)	1% (2)	0% (0)	9.1% (18)	90.9% (179)	197
Control 2 (LGMD2D)	5.6% (5)	0% (0)	2.3% (2)	7.9% (7)	92.1% (82)	89
Control 3 (hESCs)	4.4% (5)	0% (0)	7.9% (9)	12.3% (14)	87.7% (100)	114
K32del	20.9% (23)	0% (0)	0.9% (1)	21.8% (24)	78.2% (86)	110
L35P	46.6% (48)	4.9% (5)	0.9% (1)	52.4% (54)	47.6% (49)	103
R249W	28.8% (59)	0% (0)	2.9% (6)	31.7% (65)	68.3% (140)	205

OPERATOR 3 (BLINDED)

	Elongated Nuclei	Nuclei with Blebs	Deformed Nuclei	Abnormal Nuclei	Normal Nuclei	Total Number of Nuclei
Control 1 (WT hiPSCs)	4.8% (8)	1.2% (2)	0% (0)	6% (10)	94% (158)	168
Control 2 (LGMD2D)	4.2% (4)	2.1% (2)	3.1% (3)	9.4% (9)	90.6% (87)	96
Control 3 (hESCs)	2.4% (3)	1.6% (2)	1.6% (2)	5.6% (7)	94.4% (117)	124
K32del	17.4% (17)	2% (2)	3.1% (3)	22.5% (22)	77.6% (76)	98
L35P	40% (42)	4.8% (5)	1.9% (2)	46.7% (49)	53.3% (56)	105
R249W	28.4% (50)	0% (0)	1.7% (3)	30.1% (53)	69.9% (123)	176

Table S1. Scoring of nuclear abnormalities in 3D reconstructed LMNA-mutant artificial muscles, related to Figure 3 and Experimental Procedures. The three sub-tables show consistency of scoring nuclear abnormalities across different independent operators. Absolute values shown in brackets.

SUPPLEMENTAL EXPERIMENTAL PROCEDURES

Cell cultures and differentiation protocols

15 distinct hPSC lines (1 hESC + 15 hiPSC lines) and 1 primary human myoblast line were utilized in this study. Both transgene-based and transgene-free skeletal myogenic differentiation protocols of hPSCs were utilized in this work. hPSC-derived inducible myogenic cells (transgene-based; also known as HIDEMs/HEDEMs) were derived and maintained in culture as previously described (Maffioletti et al., 2015; Tedesco et al., 2012). Shef-6 cells (hpscreg.eu/cell-line/UOSe006-A) were used for hESC experiments and were kindly provided by Prof. P. Andrews (University of Sheffield, UK). The following 5 healthy donor hiPSC lines were used: 1) SBI iPSCs (System Biosciences SC102A-1N; hpscreg.eu/cell-line/SBIi006-A; kindly provided by Prof. VandenDriessche, VUB, Belgium)(Maffioletti et al., 2015); 2) in-house reprogrammed healthy control line no.4 (Tedesco et al., 2012); 3) Gibco® Episomal hiPSC Line A13777, LifeTechnologies; 4) NCRM1 (hpscreg.eu/cell-line/CRMi003-A); 5) NCRM5 (hpscreg.eu/cell-line/CRMi001-A). The following five muscular dystrophy hiPSC lines were used: 1) DMD iPSCs (deletion of exons 4-43)(Kazuki et al., 2010) were kindly provided by Prof. M. Oshimura (Tottori University, Japan); 2) LGMD2D iPSC were generated from somatic cells provided by the Munich Tissue Culture Collection (MTCC; Friedrich-Baur Institute, Munich, Germany), were previously described (Tedesco et al., 2012) and are also deposited in hpscreg.eu (UCLi005-A); 3) Three distinct *LMNA*-mutant iPSC lines were reprogrammed and provided by Cellular Dynamics International Inc. and CureCMD. Inducible myogenic cells were differentiated in DMEM, 2 % horse serum, 2 mM glutamine, 100 IU + 0.1 mg / ml penicillin/streptomycin (i.e. differentiation medium) on Matrigel-coated dishes; 1 µM 4-OH tamoxifen (Sigma, H7904) was added twice to the medium, once in proliferation medium when cells reached 90%-100% confluence and the following day in differentiation medium as previously described (Maffioletti et al., 2015; Tedesco et al., 2012). For transgene-free myogenic differentiation, 3 healthy hiPSC lines (NCRM1, NCRM5 and A13777, details above) were differentiated using a published and commercially available small molecule-based protocol (Caron et al., 2016)(Genea Biocells, Skeletal Muscle Differentiation Kit, manufacturer's instructions were followed with minor adaptations). Primary human skeletal myoblasts were

purchased from ThermoFisher (GIBCO® Human Skeletal Myoblasts, cat. no. A12555; grown and differentiated following manufacturer's instructions).

Endothelial cells and pericytes were derived from SBI iPSCs by adapting a previously published protocol (Orlova et al., 2014). Specifically, Gentle Cell Dissociation Reagent was used to create small hiPSC aggregates (STEMCELL Technologies, 07174), which were plated on Matrigel (according to (Orlova et al., 2014)) or Vitronectin (Stemcell Technologies, 07180). Cells were FACS-sorted into $CD31^+$ and $CD31^-$ fractions, obtaining comparable results to the Dynabead-based method described in the original paper. Endothelial cells were cultured as previously published (Orlova et al., 2014), whereas pericytes were cultured as described in Orlova et al. for the initial passages and then adapted to a previously described MegaCell (Sigma)-based medium optimized for skeletal muscle pericytes (Maffioletti et al., 2015; Tedesco et al., 2012) to improve expansion.

Motor neurons were differentiated from 5 hiPSC lines as recently reported (Hall et al., 2017; Stacpoole et al., 2011): a) System Biosciences SC102A-1N; b) Thermo Fisher A18945; c) Coriell ND41866*C and two in-house reprogrammed healthy control lines (Patani Lab). Derivatives from System Biosciences SC102A-1N and Thermo Fisher A18945 hiPSCs where then used in 3D co-culture experiments.

Generation of 3D artificial muscle constructs (extended version)

Fibrin gels were produced as previously published (Hansen et al., 2010) using 3.5 mg/ml human fibrinogen (TISSUCOL DUO 500, Baxter) and 10% Matrigel (BD, 356230) polymerized by adding 3 U/ml thrombin (Biopur, 10-13-1104) to the solution. The silicone posts used for gel polymerization are commercially available (EHT Technologies, GmbH Hamburg). 1×10^6 cells were used for each artificial muscle (total volume 120μl). Artificial muscles were kept in culture at 37°C with 5% CO_2 supplementing the medium with 33 μg / μl aprotinin (Sigma, A3428) to prevent fibrinogen degradation. To induce myogenic differentiation, 1 μM 4-OH tamoxifen was added 48 hours after polymerization in proliferation medium and then 24 hours later in differentiation medium. Multi-lineage artificial muscles were generated with the same method using a cell mix composed of 70% myogenic cells (7×10^5) and 30% vascular cells (6×10^4 ECs and 2.4×10^5 PCs; ratio of 1 EC : 4 PCs). Artificial muscles were cultured in a 1:1 mix of HIDEMs proliferation medium and endothelial medium A (EC-SFM basal medium, 1% Platelet-poor plasma-derived serum, 30

ng/ml VEGF, 20 ng/ml bFGF) for 48 hours then in a 1:1 mix of HIDEMs differentiation medium and endothelial medium B (Lonza, CC-3162) after the second 4-OH Tamoxifen administration. Artificial muscles were kept in culture for 10 days at 37°C with 5% CO_2, changing the medium every other day. Human fibrinogen was kindly provided by Prof. H. Redl (LBG, Vienna).

Immunostaining

For 2D immunofluorescence staining (on cells and muscle sections), samples were fixed in 4% paraformaldehyde (PFA) for 10 minutes at room temperature (RT), washed twice in phosphate buffered saline (PBS) and incubated for 30 minutes at RT with PBS - 1% bovine serum albumin (BSA) - 0.2% Triton. Samples were then incubated with 10% donkey serum for 30 minutes at RT (blocking solution) and then with the primary antibody for either 1 hour at RT or overnight (O/N) at 4°C. Unbound antibody was washed with PBS - 0.2% Triton and the cells were incubated with the secondary antibody and Hoechst 33342 for 1 hour at RT. Samples were imaged using an inverted fluorescence microscope. Artificial muscles were fixed ON in 4% PFA at 4°C, incubated for 6 hours in TBS 1X pH 7.4, 10 % FBS, 1 % BSA, 0.5% Triton X-100 at 4 °C and then with the primary antibodies in TBS 1X pH 7.4, 1 % BSA, 0.5 % Triton X-100 at 4 °C O/N. Samples were then washed over day in TBS 1X at RT and the secondary antibodies incubated in TBS 1X pH 7.4, 1 % BSA, 0.5 % Triton X-100 at 4 °C O/N. Samples were then rinsed again over day in TBS 1X at RT and subsequently imaged. For imaging, whole gels were placed on glass slides with an indentation (Carl Roth, H884.1) in fluorescence mounting medium (Dako, S3023) under a coverslip and imaged using a confocal microscope or an upright fluorescence microscope. For sectioning, fixed gels were dehydrated with a gradient of sucrose (from 7.5 % to 30 %) and then included in Tissue-Tek® O.C.T™ (4583, Sakura) for cryostat processing (10 μm thick slices). Sections were then stained as detailed for 2D cell staining and imaged using an upright fluorescence microscope (Leica). Primary antibodies used: mouse anti-myosin heavy chain (MyHC; MF20, DSHB), rabbit anti-myosin (Calbiochem, 476126), chicken anti-laminin (Abcam, ab14055), mouse anti-Lamin A/C (Novocastra, NCL-LAM-A/C), CD31 (BD, 550274). The secondary antibodies were donkey anti-mouse, goat anti-chicken, donkey anti-

rabbit (all Molecular Probes, Alexa Fluor series) used in combination with Hoechst 33342 (Fluka, B2261).

For immunohistochemistry, PFA-fixed artificial tissues were dehydrated and embedded in paraffin. Longitudinal or transverse paraffin sections (4 μm) were subjected to standard hematoxylin and eosin staining or immunohistochemistry with anti-mouse immunoperoxidase polymer (Histofine Simple Stain MAX PO, Nichirei Biosciences) and universal DAB detection kit (Ventana) after incubation with primary antibody (sarcomeric actin, Dako, M0874).

For the quantification of $CD31^+$ structures within the artificial muscle implant, tibialis anterior sections processed and stained for CD31 (to highlight endothelial cells/vessels), human LAMIN A/C (to highlight the implant) and Hoechst as described above. The area of $CD31^+$ structures within the artificial muscle was calculated with Image J, excluding the area of their lumen when present. The implant area was also calculated for each single muscle section. Data normalized to a field of $1x10^5$ $μm^2$.

DNA extraction and sequencing

QIAamp DNA Mini Kit (Qiagen, 51304) was used to extract DNA from cell pellets following manufacturer's instructions. Sequencing was performed by Source BioScience (Nottingham, UK) using the following primers: GAATCCCCTCTCCTCGCTTC (F) and GATCTTCTGGGGTGGCAGAG (R).

RNA extraction and expression analyses (RT-PCR and qRT-PCR)

RNA was extracted from cell pellets using the RNeasy Mini kit (Qiagen, 74104) following manufacturer's instructions. Trizol reagent (Life technologies, 15596-026) was used to extract RNA from artificial muscles after homogenization as detailed in the manufacturer's protocol. RNA yield and purity was assessed with a Nanodrop; variable amounts of RNA (between 500 ng and 1 μg) were then retro-trascribed to cDNA with the ImProm-II™ Reverse Transcription System kit (Promega, A3800). A PCR for the housekeeping gene GADPH was performed in order to evaluate the efficacy of the retro-transcription reaction. Quantitative Real Time-PCR to assess for the expression of myogenic factors was performed with the GoTaq® qPCR Master Mix kit (Promega, A6001) following manufacturer's directions using a BioRad CFX96 machine. For each experiment, samples were analyzed with technical duplicates/triplicates; N = 3 for all lines apart from *LMNA*-mutant and LGMD2D

hiPSCs, whose error bars in Figure 2E represent intra-experimental replicates (n = 3). Ct data were normalized using GADPH gene expression values for each sample analyzed using the ΔΔCt method (Schmittgen and Livak, 2008) and presented as mean +/- standard deviation. Please find below a list of primers used for expression analyses:

Gene	Primer sequence	Annealing Temperature
GADPH (F)	AGGTCGGTGTGAACGGATTTG	60 °C
GADPH (R)	TGTAGACCATGTAGTTGAGGTCA	60 °C
MYOD (F)	CACTCAAGCGCTGCACGTCG	64 °C
MYOD (R)	GGCCGCTGTAGTCCATCATGC	64 °C
MyHC (F)	GCATCGAGCTCATCGAGAAG	60 °C
MyHC (R)	CATACAGCTTGTTCTTGAAGGAGG	60 °C
Sgca (F; mouse)	GCCCTGCCAAGATGGGTGCTC	60 °C
Sgca (R; mouse)	GCCTTTGTGGAGCCGGAGGTC	60 °C
SGCA (F; human)	GCCTCCACTTCTGTCTTGCT	64 °C
SGCA (R; human)	CCACCAAGAAGTCACGGTCT	64 °C
DYSTROPHIN (F)	GCAAGAGCAACAAAGTGGCCTA	60 °C
DYSTROPHIN (R)	AGCTTCTTCCAGCGTCCCTCA	60 °C

GADPH: glyceraldehyde 3-phosphate dehydrogenase; MYOD: myogenic differentiation 1; MyHC: myosin heavy chain; SGCA: alpha sarcoglycan; F: forward; R: reverse.

Western blot

Proteins were extracted with cold lysis buffer (50 mM Tris HCL pH 7.4, 1 % Triton X-100, 1 mM EDTA pH 8, 150 mM NaCl in ddH_2O) with 1 mM PMSF (Thermo Scientific, 36978) and a protease inhibitor cocktail (Roche, 04693159001). Samples were homogenized on ice. Samples were incubated on ice for 30 minutes and centrifuged at 10000 rpm for 10 minutes at 4°C. Sample concentration was determined using a colorimetric reaction (BioRad, 500-0113). 30μg of proteins per sample were loaded in reducing loading buffer (Alpha Aesar, J61337) on a precast gel (4-15%; Biorad, 345-0028) and run at 100 V. Proteins were transferred onto a PVDF membrane, which was then blocked with 5% non-fat dry milk (Cell Signaling,

999S) in TBST and incubated ON at 4°C with the primary antibody. The membrane was washed three times with TBST, incubated with the secondary antibody for 1 hour at RT and rinsed with TBST. The blot signal was developed with ECL reagents (GE Heathcare, RPN2209) and imaged using a chemi-luminescence detector (Biorad, ChemiDoc MP). Anti-beta-tubulin antibody was used as loading control. The antibodies used were the following: mouse anti-MyHC (DSHB, MF20), mouse anti beta-Tubulin (Sigma, T4026), Anti-Mouse IgG (H+L)-HRP Conjugate (Biorad, 1706516).

Electron microscopy

Samples were washed twice in PBS and incubated for 10 minutes at 37 °C in 30 nM 2,3-butanedione monoxime (Sigma, cat. no. B0753). Samples were then fixed O/N at 4 °C in 0.36 % glutaraldehyde (Sigma, cat. no. G7526). Post-fixation was performed with 1 % osmium tetroxide (Science Services, cat. no. E19110) for 2 hours. Samples were then dehydrated, embedded into a glycid ether-based resin and processed in 50 nm-thick sections. Images were acquired on a Zeiss LEO 912AB microscope.

Imaging and analysis of calcium transients

Calcium transients were measured after loading hiPSC-derived artificial muscle constructs with the fluorescent Ca^{2+} indicator Fluo-4AM (ThermoFisher). A monolayer of differentiated human myotubes was used as a technical positive control for reagents and setup, whereas undifferentiated cells (same line used in the constructs) was used as negative control. Fluorescent dye loading and image acquisition was conducted in Recording Buffer (RB: Glucose 10 mM, NaCl 150 mM, KCl 4.25 mM, NaH_2PO_4 1.25 mM, $NaHCO_3$ 4mM, $CaCl_2$ 1.2 mM, $MgCl_2$ 1.2 mM, HEPES 10 mM, pH 7.4) at 37°C. Cell preparations were loaded in RB containing Fluo-4AM 5 µM and Pluronic 0.002% (w/v) for 30 minutes which was washed off with RB prior to image acquisition on the thermostat stage (at 37°C) of a Zeiss 880 CLSM. Fluorescence was excited at 488 nm, emission collected at >530 nm and images were acquired as a time series. Additions of caffeine in RB at 10 mM concentration were used to stimulate SR Ca^{2+} release via ryanodine receptors (RyR). Muscle constructs often required multiple doses of caffeine and a delayed response was also observed in some cells, possibly due to the layer of fibrin covering the myotubes and delaying interaction of caffeine with the cell. Muscle constructs were removed from

their silicone posts for imaging, which together with the handling before imaging might explain the presence of non-responding cells together with responders. Nonetheless, cells showing caffeine-induced calcium transients were detected in all 3D muscle constructs analyzed (n = 4; 3 examples shown in figure S1). Different regions of the recorded field were tracked to record the fluorescent intensity (corresponding to a small area of one cell, approximately 10% of the cell area, to avoid interfering signals from other cells in 3D). A minimum of 20 total traces was analyzed and three representative responding cells per muscle are shown in Figure S1H. The graph shows ΔF/F traces, which is the measured fluorescence relative to the average resting fluorescence of each point, before the administration of caffeine. Image analysis was conducted with ImageJ and Microsoft Excel was used to generate the graphs.

In vivo implantation studies

Adult NOD-scid-gamma immunodeficient mice (NSG) were used for transplantation assays (N = 24). Animals were anesthetized with isoflurane (in O_2 at 2 liters / minute) and the surgery was performed under sterile aseptic technique. Briefly, a longitudinal incision was performed on the skin on top of the tibialis anterior (TA) muscle to expose the muscle tissue. A 2 mm deep, 8 mm longitudinal incision was then performed to open an intramuscular pocket and a volume of muscle tissue corresponding to the hydrogel(s) needing implantation removed. Occasional bleeding was controlled with a high temperature cauterizer (Bovie Medical, AA01). Before implantation, gels were washed twice with sterile PBS and then implanted into the muscle pocket. 8μL of fibrin glue (TISSUCOL DUO 500, Baxter) was added on top of the pocket containing the implant to seal the wound and keep the artificial tissue in place. 1 or 2 gels were implanted respectively for short (1 week) or long time (2-4 weeks) analyses. The skin was sutured with 4.0 Vicryl sutures (Ethicon) and the mouse was allowed a gradual recovery from anaesthesia. Analgesia (Carprofen) was administered 40 minutes before surgery and the day after to minimize discomfort. The surgeon was not aware of the genotype or composition of the implants. All mice recovered promptly without post-operative adverse events.

For functional vascularization assays, DyLight® 594 GSL I-B4 isolectin (Vector Laboratories, DL-1207) was injected into the tail vein of NSG mice 1 week after surgical procedure (100 μg/injection). Animals were humanely killed 20 minutes after

injection, muscles dissected and then cryopreserved for further analyses. Isolectin staining was imaged on muscle sections with a fluorescent microscope (excitation wavelength: 592 nm).

Mouse muscle tissue processing

Tissues were harvested from the animals at different time points and cryopreserved as previously described (Gerli et al., 2014). Muscles were sectioned (thickness: 8 µm) using a cryostat (Leica, CM1850) and collected on polarized glass slides. Samples for RNA extraction were collected during muscle sectioning. Sections were stored at –80°C.

In vivo force assessment

Four weeks after surgery (performed as described in the "*In vivo* implantation studies section" above), muscle function was assessed for the right and left tibialis anterior (TA) muscles. Two hydrogels per NSG tibialis anterior muscle were implanted and chimeric muscles were assessed one month after implant. A total of 17 mice and 34 muscles were assessed (N = 6 muscles containing myofibres-only hiPSC-derived hydrogels, 6 muscles containing multicellular hiPSC-derived hydrogels, 5 injury-only muscles and 34 uninjured contralateral muscles). One injury-only mouse was excluded from the analysis because of technical problems during recording of muscle force. The operating surgeon was blind to the type of implanted hydrogel and muscle physiologists were blind to all groups. The muscle function assessment procedure was adapted from standard protocols (Liu M et al., 2005; Sharp PS et al., 2005) and has been previously described (Foster H et al., 2008). Mice were deeply anesthetized with Hypnorm (VetPharma, Leeds, UK) and Hypnovel (Roche, Welwyn Garden City, UK) as previously described and were carefully monitored throughout the experiment to ensure that there was no reflex response to toe pinch. The distal tendon of the TA muscle was dissected from surrounding tissue and tied with 4.0 braided surgical silk (Interfocus, Cambridge, UK). The sciatic nerve was exposed and superfluous branches axotomized, leaving the TA motor innervation via the common peroneal nerve intact. The mouse was placed on a thermopad (Harvard Apparatus, Edenbridge, UK) to maintain body temperature at 37 °C. The foot was secured to a platform and the ankle and knee immobilized using stainless steel pins. The TA tendon was attached to the lever arm of a 305B dual-mode servomotor transducer

(Aurora Scientific, Aurora, Ontario, Canada) via a custom-made steel s-hook. TA muscle contractions were elicited by stimulating the proximal part of common peroneal nerve via bipolar platinum electrodes, using supramaximal square-wave pulses of 0.02 ms (701A stimulator; Aurora Scientific). Data acquisition and control of the servomotors were conducted using a Lab-View-based DMC program (Dynamic muscle control and Data Acquisition; Aurora Scientific). Optimal muscle length (Lo) was determined by incrementally stretching the muscle using a micromanipulator until the maximum isometric twitch force was achieved. Maximum isometric tetanic force (Po) was determined from the plateau of the force-frequency relationship following a series of stimulations at 10, 30, 40, 50, 80, 100, 120, and 150 Hz. A 1-minute rest period was allowed between each tetanic contraction. For direct muscle stimulation two custom made electrodes were inserted in the mid belly of the muscle and the whole procedure was repeated using 4V stimulation. Muscle length was measured using digital calipers based on well-defined anatomical landmarks near the knee and the ankle. Results presented are the ratio of maximum tetanic force of the treated vs the untreated contralateral limb for direct muscle stimulation only, as the injury model and specific timeline appeared not to give sufficient time for innervation and assessment via sciatic nerve stimulation.

SUPPLEMENTAL REFERENCES

Foster, H, Sharp, PS, Athanasopoulos, T, Trollet, C, Graham, IR, Foster, K et al. (2008). Codon and mRNA sequence optimization of microdystrophin transgenes improves expression and physiological outcome in dystrophic mdx mice following AAV2/8 gene transfer. Mol. Ther. *16*, 1825–1832.

Liu, M, Yue, Y, Harper, SQ, Grange, RW, Chamberlain, JS and Duan, D (2005). Adenoassociated virus-mediated microdystrophin expression protects young mdx muscle from contraction-induced injury. Mol. Ther. *11*, 245–256.

Schmittgen, T.D., and Livak, K.J. (2008). Analyzing real-time PCR data by the comparative C(T) method. Nat. Protoc. *3*, 1101-1108.

Sharp, PS, Dick, JR and Greensmith, L (2005). The effect of peripheral nerve injury on disease progression in the SOD1(G93A) mouse model of amyotrophic lateral sclerosis. Neuroscience *130*, 897–910.
